# Inverse design of perimeter-controlled InAs-assisted metasurface for two-dimensional dynamic beam steering

**DOI:** 10.1515/nanoph-2022-0376

**Published:** 2022-09-02

**Authors:** Raana Sabri, Hossein Mosallaei

**Affiliations:** Department of Electrical and Computer Engineering, Northeastern University, Boston, MA 02115, USA

**Keywords:** active metasurface, high-k dielectric, perimeter-controlled architecture, two-dimensional beam scanning

## Abstract

The current commercially viable light detection and ranging systems demand continuous, full-scene, and dynamic two-dimensional point scanning, while featuring large aperture size to ensure long distance operation. However, the biasing architecture of large-area arrays with numerous individually controlled tunable elements is substantially complicated. Herein, inverse design of a perimeter-controlled active metasurface for two-dimensional dynamic beam steering at mid-infrared regime is theoretically presented. The perimeter-control approach simplifies biasing architecture by allowing column-row addressing of the elements. The metasurface consists of a periodic array of plasmonic patch nanoantennas in a metal-insulator-metal configuration, wherein two active layers of indium arsenide are incorporated into its building block. The metasurface profile facilitates wide phase modulation of ≈355° on the reflected light at the individual element level through applying independent voltages to its respective columns and rows. The multi-objective genetic algorithm (GA) for optimizing user-defined metrics toward shaping desired far-zone radiation pattern is implemented. It is demonstrated that multi-objective GA yields better results for directivity and spatial resolution of perimeter-controlled metasurface by identifying the design tradeoffs inherent to the system, compared to the single-objective optimizer. A high directivity and continuous beam scanning with full and wide field-of-view along the azimuth and elevation angles are respectively maintained.

## 1 Introduction

Fast-scanning and low-divergence optical beam generation have a major application in free space optical (FSO) communication, three-dimensional imaging, mapping, and ranging [1–6]. Metasurfaces are two-dimensional planar structures of subwavelength unit cells, whose implementation in the integrated photonics represents a great potential to overcome the limitations of mechanical beam steering by offering light-weight and compact footprints, and as a result high-speed and large field-of-view (FOV) [7–9]. For several applications, it is highly desirable to maintain dynamically tunable metasurface designs with improved scanning speeds that can effectively map the surrounding environment in real-time [10–13]. Various materials, which offer refractive index modulation in response to the external stimuli can be integrated with the metasurface building blocks in order to surmount their static operation and obtain a versatile dynamic control over their optical response. In particular, two-dimensional materials such as graphene or transition metal dichalcogenides [14–18], III–V semiconductors [19], thermo-optically [20, 21], and electro-optically tunable materials [22–24] have been widely employed for dynamic phase, amplitude, wave vector, and polarization modulation of the scattered light.

A variety of CMOS-compatible electro-optical materials including transparent conducting oxides (indium tin oxide (ITO) and cadmium oxide (CdO)) [25–28], indium arsenide (InAs) [29, 30], and gallium nitride/aluminium gallium nitride (GaN/AlGaN) [31] are recently used, whose integration with the metasurface unit cells enables enhanced light–matter interaction and wide phase modulation of light at the infrared spectral regime. Despite the substantial advantages offered by these electro-optical materials such as epsilon-near-zero (ENZ) transition, short response time (≈ns), continuous tunability, and negligible hysteresis, some of them, e.g. ITO, suffer from strong power absorption caused by the non-negligible dissipative loss. This limits the power efficiency of the ITO-integrated metasurfaces to <1% [32–34]. It is demonstrated that InAs is an alternative active material that features superior optical properties in terms of dissipative loss compared to the ITO, while maintaining the ENZ phenomenon and large variation range of complex refractive index [21, 29]. As such, it holds a great promise for integration with the geometrically-fixed metasurfaces toward obtaining an active optical response with high efficiency.

Another important step in the development of the metasurfaces for beam steering is to ensure their efficient operation over a long range as required by LiDARs on autonomous vehicles (operation range at the order of ≈100 m) and FSO communication systems (operation range over 100 km). This necessitates design of large-aperture arrays with high directivities that are composed of at least thousands of densely spaced unit cells, which should be independently controlled in order to form the desired radiation patterns. State-of-the-art in the use of photonic integrated circuits (PICs) technology for beam generation and shaping holds a great promise for integration of massive amounts of unit cells, as required for the large-aperture metasurfaces [35]. Nevertheless, the challenge lies on the complexity of the biasing network that should guarantee the independent and simultaneous control of the individual unit cells across the entire metasurface array. Indeed, for individually addressing of the elements in a generic case of two-dimensional metasurface designs, *N* × *N* array of control signals are required, which clearly increase the complexity of the biasing scheme for the dense metasurface arrays and lead to large power consumption at the interconnection of control signals and the unit cells [36, 37]. In addition, the obtained FOVs for some of the two-dimensional beam steering devices are rather small [35, 36, 38].

So far, several techniques are established to reduce the biasing network complexity and power consumption of the large scale metasurfaces and optical phased arrays (OPAs) designed for the two-dimensional beam steering. Specifically, the combination of relative phase gradient and wavelength tuning as two degrees of freedom to steer the beam along two different dimension by using an array of grating couplers is presented [39, 40]. Despite the simplified biasing network, this approach suffers from large spectral bandwidth, required for the wavelength tuning, which gives rise to noise effect, inefficient power distribution within the bandwidth, and limited FOV. In another work, design of serpentine OPA based on serially interconnected array of grating waveguides for addressing the control complexity, power consumption, and optical efficiency of large scale OPAs is demonstrated. The maximal achievable FOV in this case is at the range of 36° × 5.5° [41]. Several studies have considered the column-row addressing of the elements to surmount the complex biasing network of the two-dimensional rectangular arrays [42–45]. In this technique, the individual elements of an *N* × *N* array can be independently addressed by the superposition of control signals that are applied to the respective columns and rows of the array. This approach avoids the complex per-element control schemes to address *N*^2^ spots and reduces the number of control signals to 2*N*. The row-column addressing mechanism implemented earlier, rely on tuning of the input guided wave by using perimeter-controlled phase shifters to alter the phase and amplitude of the constituent elements of the conventional OPAs. However, for the metasurfaces, whose excitation are by illuminating wave into their sub-wavelength scatterers, the row-column addressing of the biasing signals rather than the input carrier feed should be considered [46].

Recently, considerable efforts have been devoted for inverse design of the large-scale metasurfaces in the array level, which is implemented to optimize the array architecture of the metasurfaces within high-dimensional input parameter space [47]. For this purpose, deep neural networks [48], adjoint variable method [49, 50], genetic algorithm [51], and machine learning techniques [52] are proposed, which aim at obtaining the on-demand optical functionality by optimizing the surface morphology of the nano-scale elements. However, the topology-based optimization of the metasurface building blocks does not satisfy the requirements toward achieving the desired functionalities of the active metasurfaces such as dynamic beam steering. As such, there is an intense interest for an inverse deign objective that is applicable to the dynamically tunable metasurfaces, with the goal of finding the optimal functional characteristics such as amplitude and phase distribution across the entire array in response to their input [53, 54]. This approach allows for co-optimization of the scattering metrics in the array level toward obtaining the desired array performance objective. The array-level inverse design of the active metasurfaces exploits the tunability of the individual active unit elements that is facilitated by the independent per-element biasing. This introduces a computational complexity and time cost to the optimization problem due to the high-dimensionality of the input space corresponding to the external stimulus of the constituent elements of the large-scale array. To surmount these obstacles, the optimization of the scattering properties of the light in the super cell level, whose periodic repetition arranges the entire large-aperture array, is proposed that substantially reduces the computational cost of the large-scale inverse design problem [55].

Various optimization strategies for inverse deign of the active large-area metasurfaces can be used, which are broadly categorized into local, global, and multi-objective approaches [56]. The local and global optimization algorithms can be employed to minimize the user-defined objective functions and provide an optimal design in terms of predetermined performance criteria. The optimal design is obtained through exhaustive scanning within the input parameter space that is limited to finite ranges in order to incorporate the constraints such as fabrication feasibility, material selection, and size, weight, and power considerations. Global optimization algorithms have advantage over the local optimizers in their attempt to find the true global minimum and not getting stuck in local minima, while optimizing for ill-suited problems. Nevertheless, these conventional algorithms only seek for a single cost function and provide a single solution without yielding any additional information on the design objective tradeoffs inherent to the system. Multi-objective evolutionary optimization algorithms, on the other hand, provide an intuition about the tradeoffs between the multiple competing design objectives and produce a solution space called Pareto optimal front [57, 58]. This is particularly relevant for identifying the ultimate performance of the active metasurfaces with large degrees of freedom in their functional characteristics in response to the external stimulus.

Here, we adopt the computational framework based on the multi-objective GA evolutionary optimizer for the inverse design of perimeter-controlled tunable metasurface for two-dimensional dynamic beam steering. The perimeter-control architecture offers a drastically simplified biasing scheme for the large-aperture arrays by enabling the column-row addressing of the control signals, while allowing for phase tuning at individual element level. Exploiting the multi-objective GA can be highly beneficial for the iterative optimization of the user-defined objective functions due to its capability in identifying the Pareto optimal solutions and considering the tradeoffs between the competing objectives. The objective functions that analytically describe the desired metasurface performance for two-dimensional beam steering are considered to be the phase profiles of the unit cells across the reflectarray and the maximum achievable directivity at the desired steering angles. We have shown that the inverse design approach exploiting multi-objective GA succeeds in obtaining the non-intuitive array amplitude and phase profiles that significantly enhance the directivity in comparison to the single-objective optimization algorithm. To proof of concept, we consider the dual-gated plasmonic patch nanoantennas in metal–insulator–metal (MIM) configuration that are integrated with InAs active layer as a building block of the metasurface. In order to render the perimeter-control architecture for the column-row biasing, the patch nanoantennas at the same columns as well as the InAs active layers at the same rows are electrically connected via the strip electrodes, while the columns and rows are electrically isolated from another. In this case, the optical response of an arbitrary element (the reflection amplitude and phase) is a function of voltages applied to the respective column and row of the metasurface where that specific element is located. We have shown that maximal achievable FOV by the perimeter-controlled metasurface reflectarray enabled by inverse design method is maintained in the range of (*θ*_s_, *φ*_s_) = 40° × 360° at mid-infrared spectral regime. The FOV along the elevation direction can be further increased by fixing the azimuth angles into 0°, 90°, 270°, and 360°.

## 2 InAs-assisted active unit cell design

Active spectral tuning of the optical antenna resonances constitutes one of the most efficient ways to achieve local modulation of the scattered light. One of the most common approaches to actively tune the optical properties of the metasurfaces is through integrating the semiconductor materials into their building blocks and modulating their free carriers by means of electrical gating. Transparent conducting oxides such as ITO, aluminum zinc oxide (AZO), and CdO have been widely used for designing the dynamically tunable optical modulators at the telecommunication band [26, 59, 60]. The achievable changes in the optical response of metasurfaces integrated by TCOs can be further enhanced by operating at the ENZ point. Nevertheless, it has been shown that metasurfaces integrated with active materials such as ITO demonstrate low efficiency due to the high absorbance of the incident light within the extremely thin accumulation layer of under-biased ITO [61]. The low-doped InAs active layer can be alternatively used for designing the high efficiency tunable metasurfaces, since it features substantially smaller dissipation loss compared to ITO. In addition, the effective mass of InAs is lower than ITO, which ensures large variation range of the plasma frequency for the same levels of the charge carriers [29].

**Figure 1:**
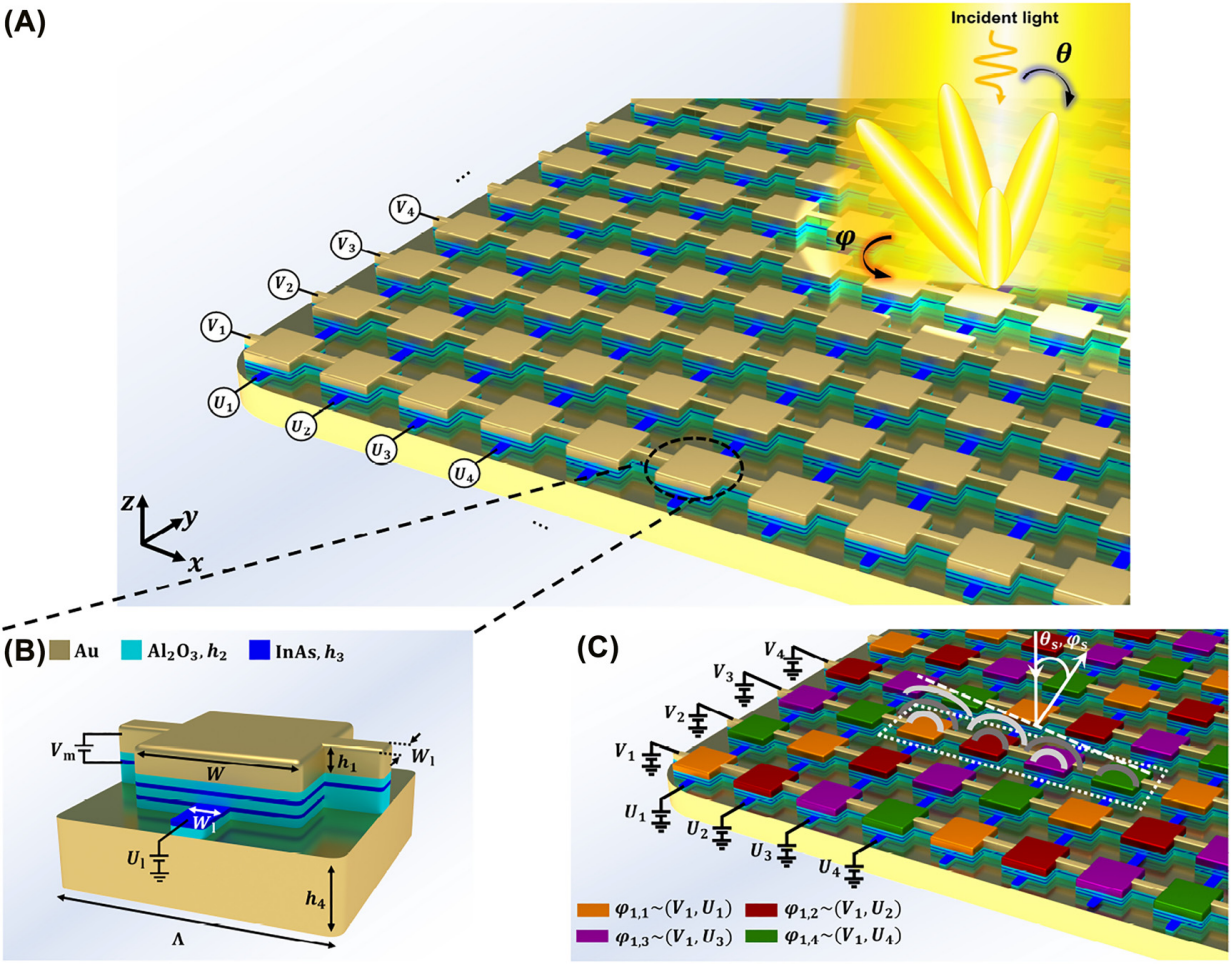
Perimeter-controlled metasurface for two-dimensional dynamic beam steering. (A) Pictorial illustration of the perimeter-controlled plasmonic reflectarray under the normal illumination of the incident wave for two-dimensional beam steering. (B) The configuration of the plasmonic unit cell. Constitutive layers from top to bottom: Au square patch (*h*_1_ = 23 nm, *W* = 650 nm, and electrode *W*_l_ = 150 nm), alumina dielectric spacer (*h*_2_ = 15 nm), InAs active layer (*h*_3_ = 10 nm), alumina dielectric layer (*h*_2_ = 15 nm), InAs active layer (*h*_3_ = 10 nm), alumina dielectric layer (*h*_2_ = 15 nm), and Au back reflector (*h*_4_ = 200 nm). The Au electrodes connect the patch nanoantennas in the same row along *x* direction and the InAs electrodes connect the bottom InAs layers in the same column along the *y* direction. The biasing configuration allows for independent biasing of columns and rows by application of the external bias voltages of *U* and *V*, respectively. The reflectarray consists of 9 × 9 elements with the inter-element spacing of Λ = 1033 nm. (C) Conceptual illustration of the row–column addressable elements. The colored patches represent the elements with different phase distribution required for beam steering toward the angles (*θ*_s_, *φ*_s_).

The schematic of the perimeter-controlled InAs-assisted tunable metasurface is pictorially illustrated in Figure 1(A). As a proof of concept platform, we consider the dual-gated MIM configuration that is integrated with two layers of InAs as the unit cell, whose periodic repetition arranges the entire metasurface reflectarray. Each unit cell consists of a rectangular gold (Au) nanoantenna over the stack of alumina/InAs/alumina/InAs/alumina heterostructure, and the entire composite structure is backed by an optically thick gold substrate, serving as a back reflector (Figure 1(B)). To facilitate the column–row control of the metasurface reflectarray, two InAs active layers are employed that enable dual-gated biasing and ensure the minimal mutual coupling effects between the accumulation/depletion layers. The strip electrodes intersecting with the patch nanoantenna as well as InAs layers along the *x* and *y* directions enable the formation of equipotential rows and columns for yielding the perimeter-control architecture, respectively. The equipotential rows are biased by applying the DC voltages to Au and top InAs electrodes, while the equipotential columns are independently addressed by using the bottom InAs layer and the back reflector as contact electrodes (Figure 1(B)). It should be noted that the proposed biasing configuration allows for phase tuning in the individual element level through addressing the corresponding column and row, where that specific element is located (Figure 1(C)). The gate-dielectric material is selected to be alumina, since it exhibits relatively high breakdown field and good thermal stability. Moreover, it has a much simpler fabrication process in comparison to the nanolaminate gate-dielectric layers such as HAOL [25]. For the optical simulations, that are carried out with the finite difference time domain (FDTD) solver of Lumerical, the electric permittivity of the alumina is extracted from the experimental data in Ref. [25] at mid-infrared range, whereas the InAs and gold layers are characterized by the Drude dispersion permittivity model as $\epsilon =\epsilon_{\infty }-\omega_{p}^{2}/\left(\omega^{2}+i\omega \Gamma \right))$. The parameters *ϵ*_∞_, *ω*_*p*_, *ω*, and Γ respectively imply to the high-frequency permittivity, the plasma frequency, the angular frequency of excitation, and the collision frequency. The plasma frequency of InAs is related to its charge carrier concentration (*N*) as $\omega_{p}=\sqrt{Ne^{2}/\epsilon_{0}m^{*}}$, where *e* is the charge of electron, *ϵ*_0_ is the permittivity of free space, and m* = 0.0394*m*_*e*_ is the effective mass of electron in which *m*_*e*_ is the electron rest mass. The effective mass of InAs is orders of magnitude smaller than that of the ITO (with $m_{\text{ITO}}^{*}=0.35m_{e}$) [25], as a result, InAs active layer offers a large variation range of plasma frequency for the same carrier concentration in comparison to the ITO. Throughout this paper, the dispersion parameters of InAs are adjusted to *ϵ*_∞_ = 12, Γ = 2 × 10^13^ rad/s, and the background doping densities of the carriers in the unbiased InAs layers are considered to be *N* = 1 × 10^19^ cm^−3^, that are consistent with the experimental data reported in the recent studies [21, 29]. The constant parameters in the Drude model of Au are fixed at *ϵ*_∞_ = 1.53, Γ = 2*π* × 17.64 THz, and *ω*_*p*_ = 2*π* × 2.069 PHz. The geometrical parameters of the MIM unit cell are selected in order to maximize the phase swing and the amplitude level at the operating wavelength of *λ* = 3.381 μm by tuning the external DC voltage applied to the unit cell, as defined in the caption of Figure 1(B).

The fabrication of the dual-gated InAs-assisted unit cell can be performed by atomic layer deposition (ALD) and electron beam lithography (EBL). The Au back reflector can be deposited on top of the silicon wafer by electron beam evaporation [22]. The alumina gate-dielectric can be grown by the ALD over the Au substrate. The active doped InAs layer can be formed by molecular beam epitaxy (MBE) [33]. Additional ALD and MBE processes are required for growing the alternative alumina and InAs layers. The topmost Au layer should be fabricated by electron beam evaporation. For patterning the cross-shaped layers, first the entire structure should be coated by a sufficiently thick photoresist. The desired pattern then can be developed by EBL [22, 62]. Finally, the photoresist must be removed in acetone.

**Figure 2:**
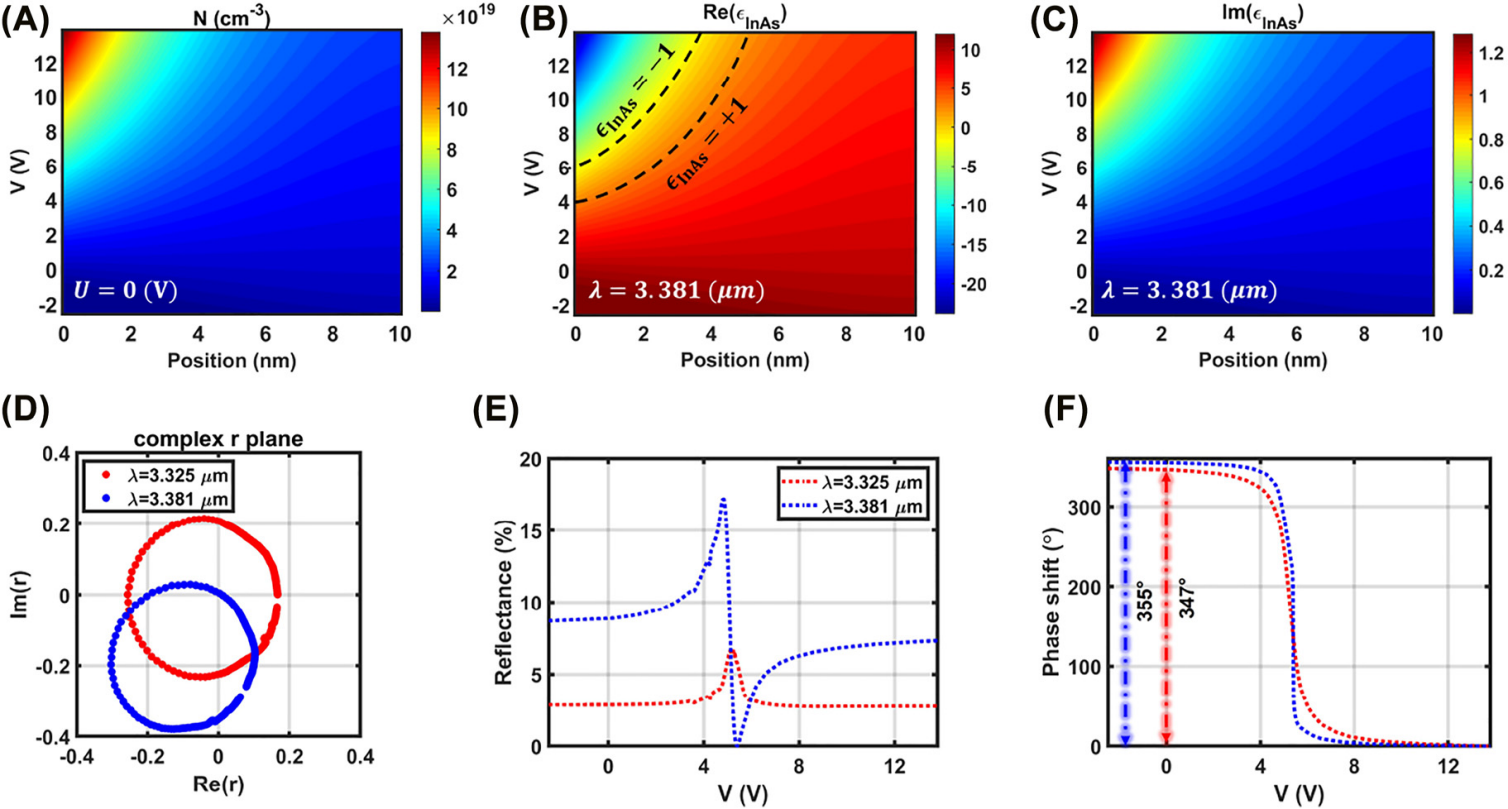
Electro-static and dynamic response of the metasurface to the external bias voltage. (A) Spatial distribution of the carrier density within the InAs active layer as a function of applied bias voltage of *V*, when *U* is set to 0 V. (B) Real and (C) imaginary parts of the InAs permittivity as functions of the bias voltage and position at the operating wavelength of 3.381 μm. The dashed contours in (B) specify the boundaries of the ENZ region. (D) The complex reflection coefficient of the plasmonic metasurface by varying the bias voltage *V* calculated at two operating wavelengths of *λ* = 3.325 μm (red curve) and *λ* = 3.381 μm (blue curve). The corresponding (E) reflectance and (F) phase response of the metasurface under the application of bias voltage of *V* when *U* is adjusted to 0 V. The large phase span of 355° can be maintained.

In order to rigorously study the carrier dynamics within the InAs active layer under the application of bias voltages, Lumerical device simulations are carried out, that self-consistently solve the Poisson and drift-diffusion equations. The dual-gated InAs-assisted metasurface is biased by applying two sets of independent voltages *V* and *U* that are respectively applied to the top and bottom electrodes. For the device simulations, InAs is modeled as the degenerately doped semiconductor with the background doping density of *N* = 1 × 10^19^ cm^−3^, while the DC permittivity, the bandgap energy, and the electron mobility are respectively adjusted to *ϵ*_DC_ = 14.6, *E*_bg_ = 0.36 eV, *μ* = 32,500 cm^2^ V^−1^ S^−1^ [21, 29]. Figure 2(A) represents the spatial distribution of the charge carrier within the 10 nm top InAs layer, when the bottom bias voltage is adjusted to *U* = 0 V and *V* is varying in the range of −2.5 to 13.8 V. For the bias voltages within the range −2.5 to 0.6 V, the depletion layer is formed at the interface of top InAs layer with alumina and upon increasing the bias voltage *V* beyond 0.6 V, the electrons are accumulated at the interface. It should be noted that the upper limits for the biasing voltages, calculated as 13.8 V, are defined by the breakdown field strength of the alumina gate-dielectric that is reported as 7.4 MV/cm [25]. In addition, our Lumerical device simulations reveal that upon further reduction of the bias voltage to <−2.5 V, the holes are accumulated at the InAs/alumina interfaces (see Supplementary Material Section S1). The contribution of the holes introduces additional loss into the system and results in decrement of the real part of the InAs electric permittivity, which leads to a lower reflectivity. To avoid the accumulation of the holes at the InAs/alumina interfaces, the lower bounds of the bias voltages are fixed at −2.5 V. The charge carrier accumulation/depletion is formed within a small portion of the InAs layer, which is located directly beneath the gate electrodes and the charge carriers exponentially decay by moving away from the InAs/alumina interfaces. As such, the electro-optical modulation of the permittivity is restricted to the nanometer-thin accumulation or depletion layers.

Demonstrated in Figure 2(B) and (C) are the spatial distribution of the real and imaginary parts of the electric permittivity of InAs as functions of the DC bias voltage. They are obtained at the operating wavelength of *λ* = 3.381 μm by linking the carrier dynamics of the InAs layer to its optical parameters through the carrier-dependent Drude dispersion model. The optical characteristics of InAs can be dynamically tuned form positive electric permittivity (dielectric nature) to negative permittivity (plasmonic nature) by increasing the bias voltage. Upon transitioning the relative permittivity form positive to negative values, the ENZ phenomenon (|Re(*ϵ*_InAs_)| < 1) occurs, which results in significant enhancement of light–matter interaction. The boundaries of the ENZ region are marked by the dashed lines in Figure 2(B). By increasing the bias voltage and further accumulation of the carriers at the InAs/alumina interface, the imaginary part of the InAs relative permittivity grows larger and proportionally the intrinsic material loss increases, as indicated in Figure 2(C). This dynamic transition of the relative permittivity between the optically dielectric and optically plasmonic regimes is the physical operating mechanism of our proposed electro-optically tunable metasurface.

Once the complex dielectric permittivity of InAs as a function of DC bias voltages is modeled, the optical response of the active metasurface under the normal illumination of transverse magnetic (TM) polarized plane wave (electric field along the *y* direction in Figure 1(A)) can be calculated. Figure 2(D)–(F) plot the reflection from the metasurface as a function of applied bias voltage at two operating wavelengths *λ* = 3.325 μm (red curves) and *λ* = 3.381 μm (blue curves). The results are calculated when the bias voltage *V* is varying from −2.5 to 13.8 V, while *U* is fixed at 0 V. As it can be seen, both the phasor diagrams almost cover all four quadrants of the complex *r* −plane, ensuring a substantially wide phase modulation (Figure 2(D)). The operating wavelength of *λ* = 3.325 μm corresponds to the maximum achievable phase span of 355° that is obtained at the cost of large amplitude variation |*r*_max_| − |*r*_min_|/|*r*_max_| = 99.9% (Figure 2(E) and (F)). Due to the strong resonant dispersion of the quasi-static metasurfaces, by straying away from the resonant wavelength, the achievable phase shift drops, which is followed by a reduction in the amplitude modulation, as well. Specifically, at the operating wavelength *λ* = 3.325 μm, the phase pickup of 347° is achieved and the amplitude variation is reduced to 58% (Figure 2(E) and (F)). It is worth mentioning that due to the smaller dissipative loss of InAs in comparison to ITO, both the reflectance and phase span of InAs-integrated plasmonic metasurface are considerably larger than its ITO-assisted counter part. It has been shown that in the active metasurfaces based on ITO the maximum achievable phase span is ≈300°, which is obtained at the cost of strong amplitude variation with reflectance level <1% at telecommunication C-band [25]. In order to use ITO as active layer in mid-infrared range (around 3 μm), its background doping density should be increased up to $\approx 10^{20}$ cm^−3^ for ensuring ENZ transition. This would give rise to imaginary part of ITO permittivity and will further reduce its efficiency.

**Figure 3:**
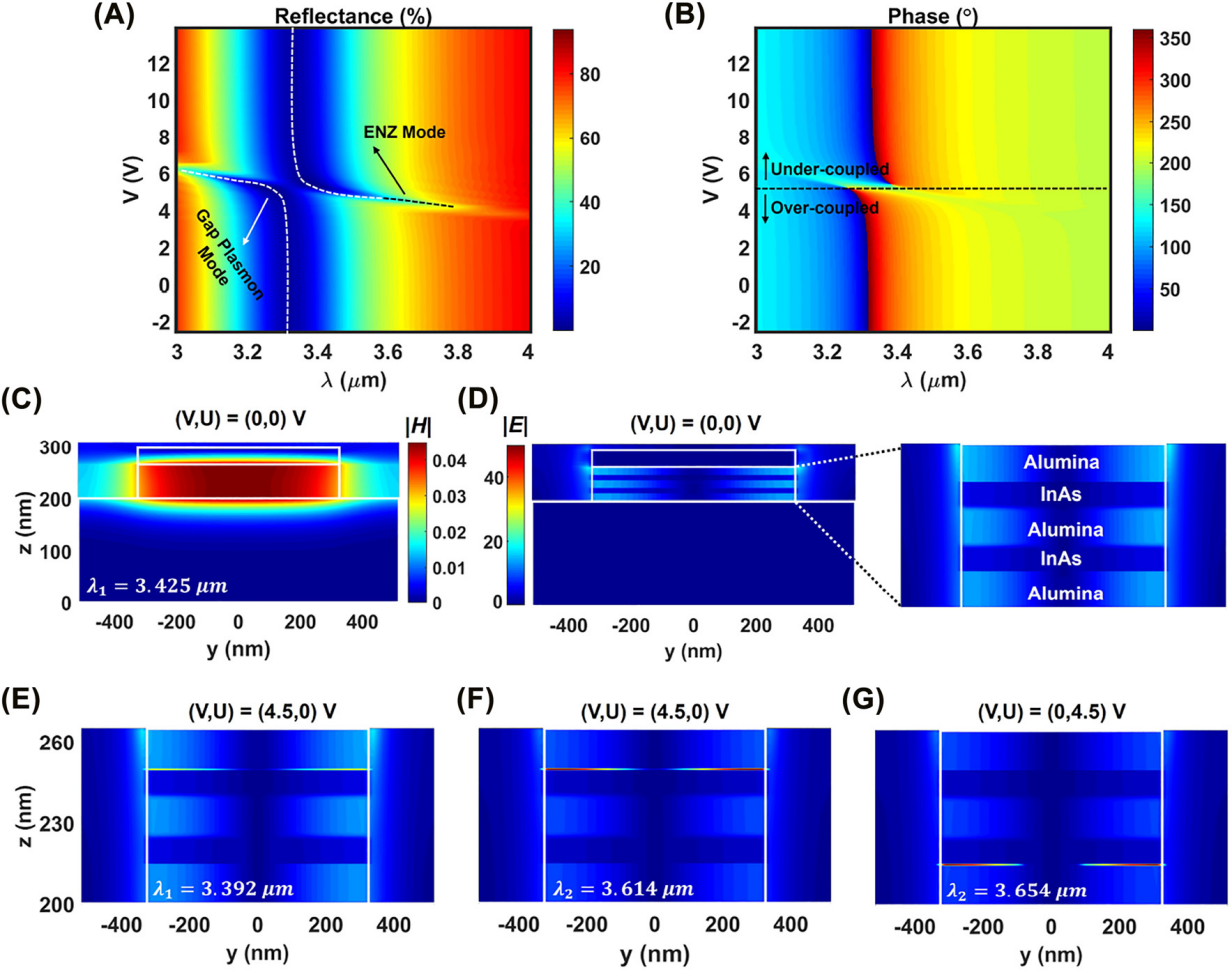
Spectral response of the under-biased metasurface and the near-field distribution. (A) Reflectance and (B) phase distributions of the reflected light as functions of the incident wavelength and applied bias voltage. The spectral overlap between two modes is observed around the operating wavelength of ≈3.38μm. The dashed lines in (A) and (B) respectively mark the spectral position of the resonances that shift to shorter wavelengths by increasing the bias voltage and the applied bias voltage corresponding to the critical coupling point between over-coupled and under-coupled resonant regime. Distributions of the total (C) magnetic and (D) electric fields within the nanoantenna heterostructure at no-bias at the wavelength of 3.425 μm. The right figure of (D) represents the zoomed-in image of electric field inside the alumina/InAs/alumina/InAs/alumina stack. Electric field distribution within the alumina/InAs/alumina/InAs/alumina stack of nanoantenna heterostructure for wavelength and bias voltage combinations of (E) *λ* = 3.392 μm, (*V*, *U*) = (4.5, 0) V, (F) *λ* = 3.614 μm, (*V*, *U*) = (4.5, 0) V, and (G) *λ* = 3.654 μm, (*V*, *U*) = (0, 4.5) V.

An additional perspective on the performance of the InAs-assisted plasmonic metasurface can be obtained by studying the spectral amplitude and phase profiles of the reflected wave from the metasurface in response to the external bias voltage. Figure 3(A) and (B) illustrate the reflection amplitude and phase as functions of the incident wavelength and the DC bias voltage *V*, when *U* is adjusted to 0 V. From the reflectance profile (Figure 3(A)) it can be observed that increasing the applied bias voltage from −2.5 V up to ≈6 V results in the spectral shift of the geometrical resonance toward the shorter wavelengths due to the reduction in the real part of the InAs permittivity. In addition, the significantly reduced loss of the InAs layer compared to ITO, leads to emergence of the intriguing ENZ resonance for the bias voltages >4 V at the longer wavelengths. This bias voltage corresponds to the point where the ENZ condition within the InAs accumulation layers happens (see Figure 2(B)). By an spectral overlap between the two resonant modes associated to the metasurface reflectarray, an operating regime that simultaneously features high amplitude level and wide phase pickup can be maintained (Figure 2(E) and (F)). From Figure 3(B) one can see that, at the fixed wavelength of 3.381 μm, each resonant mode imparts almost 180° phase shift to the reflected light by changing the bias voltage. The phase pickup associated to each resonance is acquired, since the operating regime of the metasurface shifts between the under- and over-coupled resonant regimes upon formation of ENZ region at the InAs layer [33].

In order to provide a further insight on the performance of the active metasurface under the application of electrical bias, the distributions of the electromagnetic fields within the metasurface unit cell are calculated. For this purpose, different combinations of the bias voltages are applied to the dual-gated metasurface and the results are obtained at its resonant wavelengths as plotted in Figure 3(C)–(G). From the spatial distribution of the near-field at the unbiased metasurface one can see the strong localization of the magnetic field (Figure 3(C)) and the formation of electric field node (Figure 3(D)) at the center of the structure that are the manifestations of the magnetic dipole resonant mode (gap plasmon resonance). The zoomed-in view of the electric field within the alumina/InAs/alumina/InAs/alumina spacer is shown at the right side of Figure 3(D) at operating wavelength 3.425 μm in the absence of any bias voltage. Upon applying the bias voltage combination of (*V*, *U*) = (4.5, 0) V, the charge carriers are accumulated at the interface of top InAs/alumina layers at resonant wavelength of *λ* = 3.392 μm that corresponds to the gap plasmon mode (Figure 3(E)). By changing the operating wavelength to the *λ* = 3.614 μm, which is associated to the ENZ mode, the confinement of the electric field is enhanced at the top InAs/alumina interface compared to gap plasmon resonance (Figure 3(F)). This can be attributed to the operating regime of the resonator that remains at the over-coupled mode (lower power absorption). To further investigate the nature of the ENZ resonant mode excited at the longer operating wavelength for the bias voltages larger than 4 V, FDTD simulations are performed to calculate the response of the metasurface to the variations of the structural and material properties and the results are shown in Supplementary Material Section S2. It is worth to note that the large electric field enhancement within the InAs active layers is due to the ENZ transition of their relative permittivity and the continuity of the normal component of the electric displacement at the interfaces. To illustrate that the metasurface elements positioned at different columns and rows of a reflectarray can be independently controlled via simultaneous application of the voltage to their gate electrodes, we have calculated the near-field by adjusting the bias voltage combination into (*V*, *U*) = (0, 4.5) V and *λ* = 3.654 μm. From the result indicated at Figure 3(G), it is observed that the accumulation layer is only formed within the bottom InAs/alumina interface, while the interface of top InAs layer with the alumina gate-dielectric is not affected by the bottom gate voltage. The independent control of the accumulation layers can be also verified from Figure 3(F) that shows the bottom active layer remains unchanged regardless of the bias voltage application to the top electrode and accumulation of the charge carries within top active layer. As such, the carrier-induced electrorefraction inside the top and bottom InAs active layers can be independently and simultaneously controlled by the bias voltages without a crosstalk.

## 3 Perimeter-controlled reflectarray design for tunable two-dimensional beam steering

The conventional control architecture of the metasurface arrays is based on the individual addressing of their constituent unit cells by the external stimuli such as voltage, temperature, and magnetic fields. As required for long-range operation, notably LiDAR on autonomous vehicles, remote-sensing, and FSO communication, the metasurfaces should have large aperture size to provide enhanced directivity. For the large-area metasurfaces with increased number of array elements, the simultaneous and independent biasing becomes very challenging that leads to dramatic complexity of the control network architecture. This is because for an array consisting of an ensemble of *N* × *N* elements, *N*^2^ independent control signals are needed. On the other hand, the perimeter-control architecture offers a simplified control approach by reducing the number of biasing signals into 2*N* that are, respectively, applied to the metasurface columns and rows. In this case, the metasurface element *p*, that is located at the column *l* and row *m*, is driven by two sets of bias voltages *U*_*l*_ and *V*_*m*_ that are applied to its respective column and row. As such, the biasing voltage of the *p*th element *V*_*p*_ is dependent to the voltage combinations *V*_*m*_ and *U*_*l*_ and can be represented as *V*_*p*_ = *f*(*V*_*m*_ + *U*_*l*_) [46]. To establish a general analytical solution for the far-field radiation pattern of the quasi-static reflectarray, we consider a linear array composed of *N* × *N* identical elements that are positioned along the *x* and *y* axes with the inter-element spacing of Λ_*x*_ and Λ_*y*_, respectively. Each individual element is driven by an external DC bias voltage. The radiated electric field in the far-zone of such reflectarray can be expressed as [63].

(1) $$\begin{array}{ll} E^{ff}\left(\theta ,\varphi \right) & =\underset{l}{\sum }\underset{m}{\sum }r_{lm}\exp\left(i\psi_{lm}\right)\\ & \;\times \exp\left[ilk\Lambda_{x}\sin\left(\theta \right)\cos\left(\varphi \right)\right.\\ & \;\left.+imk\Lambda_{y}\sin\left(\theta \right)\sin\left(\varphi \right)\right] \end{array}$$

where *l* and *m* respectively numerate the array columns and rows along the *x* and *y* axes, *k* is the free space wave vector, and *r*_*lm*_ exp(*iψ*_*lm*_) represents the complex reflection coefficient of the *pth* element located in column *l* and row *m*. In our analysis, we assume a constant and identical amplitude for all metasurface elements (*r*_*lm*_ = *r*) that allows to show the manifestations of the perimeter-control architecture. In addition, we consider that the relative phase of each element is changing with the variations of the bias voltage and can be expressed as *ψ*_*p*_(*V*_*p*_) ≡ *F*(*V*_*p*_) = *F*[*f*(*V*_*m*_ + *U*_*l*_)]. By a valid assumption of *F*[*f*(*V*_*m*_ + *U*_*l*_)] = *V*_*m*_ + *U*_*l*_, which is typically obtained in experiments [46], the reflected electric field from the perimeter-controlled tunable reflectarray at the far-zone can be described as

(2) $$\begin{array}{ll} E_{\text{perim}}^{ff}\left(\theta ,\varphi \right) & =r\times \underset{l}{\sum }\underset{m}{\sum }\exp\left(iV_{l}+iU_{m}\right)\\ & \;\times \exp\left[ilk\Lambda_{x}\sin\left(\theta \right)\cos\left(\varphi \right)\right.\\ & \;\left.+imk\Lambda_{y}\sin\left(\theta \right)\sin\left(\varphi \right)\right] \end{array}$$

Equation (2) implies that the far-field radiation pattern of the perimeter-controlled reflectarray can be calculated by linear combinations of two independent vectors along the orthogonal directions of the array being its columns and rows. These independent vectors are associated to the phase gradient across the reflectarray rows and columns [46].

In order to dynamically control the emission direction of the beam steering reflectarray, a progressive phase delays of Δ*ψ*_*x*_ and Δ*ψ*_*y*_ should be respectively enforced between the immediately adjacent elements along the *x* and *y* directions through adjusting the bias voltages such that *ψ*_*x*_ = *l*Δ*ψ*_*x*_ and *ψ*_*y*_ = *m*Δ*ψ*_*y*_. Therefore, the far-zone electric field distribution above this finite quasi-periodic array, under the assumption of local periodicity, which is defined by Eq. (1) can be rewritten in the following form

(3) $$\begin{array}{ll} E^{ff}\left(\theta ,\varphi \right) & =r\times \underset{l}{\sum }\exp\left[il\left(k\Lambda_{x}\sin\left(\theta \right)\cos\left(\varphi \right)+\Delta \psi_{x}\right)\right]\\ & \;\times \underset{m}{\sum }\exp\left[im\left(k\Lambda_{y}\sin\left(\theta \right)\sin\left(\varphi \right)+\Delta \psi_{y}\right)\right] \end{array}$$

From the analogy between the Eqs. (2) and (3), it is trivial to show that the expression for the far-zone radiation of the perimeter-controlled array is identical to that of the individually-controlled array elements $\left(E_{\text{perim}}^{ff}\equiv E^{ff}\right)$, that is yielded when *V*_*l*_ = *l*Δ*ψ*_*x*_ and *U*_*m*_ = *m*Δ*ψ*_*y*_. This reveals that by adjusting the phase gradient along the rows and columns of the metasurface that are proportional to their corresponding bias voltages, the requirement toward beam steering with the individually biased elements is satisfied and the reflected beam can be dynamically directed to desired points. By considering a constant amplitude for all the elements, it is straightforward to rewrite Eq. (3) as

(4) $$\begin{array}{cc} E^{ff}\left(\theta ,\varphi \right) & =r\times \left[\frac{1}{N_{x}}\frac{\sin\left(\frac{N_{x}}{2}\left(k\Lambda_{x}\sin\left(\theta \right)\cos\left(\varphi \right)+\Delta \psi_{x}\right)\right)}{\sin\left(\frac{1}{2}\left(k\Lambda_{x}\sin\left(\theta \right)\cos\left(\varphi \right)+\Delta \psi_{x}\right)\right)}\right]\\ & \;\times \left[\frac{1}{N_{y}}\frac{\sin\left(\frac{N_{y}}{2}\left(k\Lambda_{y}\sin\left(\theta \right)\sin\left(\varphi \right)+\Delta \psi_{y}\right)\right)}{\sin\left(\frac{1}{2}\left(k\Lambda_{y}\sin\left(\theta \right)\sin\left(\varphi \right)+\Delta \psi_{y}\right)\right)}\right] \end{array}$$

wherein *N*_*x*_ and *N*_*y*_ refer to element number along the *x* and *y* directions, respectively. The direction of the main beam, identified by *θ*_s_ and *φ*_s_ can be attained by calculating the maxima of Eq. (4) as

(5) $$\begin{array}{c} k\Lambda_{x}\sin\left(\theta \right)\cos\left(\varphi \right)+\Delta \psi_{x}=2m_{1}\pi \;m_{1}=0,\pm 1,\pm 2,\ldots \\ k\Lambda_{y}\sin\left(\theta \right)\sin\left(\varphi \right)+\Delta \psi_{y}=2m_{2}\pi \;m_{2}=0,\pm 1,\pm 2,\ldots \end{array}$$

In this context, the steering angles of *θ*_s_ and *φ*_s_ are referred to as anomalous reflection angles (as opposed to specular reflection angles), while *m*_1_ and *m*_2_ enumerate the anomalous diffraction orders. Equations (1)–(5) imply that by judicious adjustment of the voltages applied to the columns and rows, the desired phase gradient, and associated beam steering can be maintained. It should be noted that derivation of Eqs. (1)–(5) relies on the assumption of a uniform amplitude across the metasurface. Although the phase-only modulation of the light with reflective metasurfaces has been demonstrated [64, 65], it is typically challenging to obtain a uniform amplitude by quasi-static metasurfaces, whose operation is based on the switching between the over- and under-coupled resonant regimes. The amplitude variation with the applied voltage gives rise to the undesired sidelobe levels in beam steering metasurfaces [55].

To surmount such limitations that are caused by the non-ideal unit cell performance, we use the optimization-driven inverse design framework to define the optimal values of the DC bias voltages assigned to the columns and rows in order to direct the reflected beam into the target angle with the maximal directivity. For this purpose, we adopt the multi-objective evolutionary optimization based on GA that is developed on the optimization toolbox of MATLAB, to compute the bias voltage configuration of perimeter-controlled reflectarray. Due to the covarying amplitude and phase profiles of the unit cells, the simultaneous optimization of performance metrics are considered, and their tradeoffs are taken into account through defining the interplay of the objective functions. For the proof-of-concept, we have considered an ensemble of 9 × 9 array; nevertheless, the design principle is generic and can be applied across all the active metasurface platforms with arbitrary element count. The crosstalk effects between the adjacent unit cells in the array configuration are studied to investigate the influence of the electrical biasing upon each element on its neighboring unit and the results are presented in Section S3 of the Supplementary Material. In the inverse design of the perimeter-controlled beam steering metasurface by multi-objective optimizer, the ultimate goal is to calculate on-demand spatial distribution of the radiation pattern with high directivity that points into the desired steering angles of *θ*_s_ and *φ*_s_ at the operating wavelength of *λ*. Therefore, the objective functions are selected to be the phase gradient across the metasurface array (Δ*ϕ*) and the maximum directivity at the major lobe (*D*_0_), which should be simultaneously optimized by evolving over multiple generations. Thus, the multi-objective optimization problem can be written in the form of

(6) $$\begin{array}{cc} & \underset{P}{minimize}\;\Delta \phi \left(P,\lambda \right)\\ & \underset{P}{maximize}\;D_{0}\left(P,\lambda \right) \end{array}$$

***P*** is the matrix containing the DC bias voltages applied to the columns and rows as an input of the algorithm. The lower and upper bounds of the bias voltages are adjusted to −2.5 and 13.8 V, which are dictated by the electrostatic simulations (see Supplementary Material Section S1). To evaluate the objective functions in Eq. (6), the far-field radiation pattern of the perimeter-controlled metasurface, demonstrated by Eq. (2), is calculated based on the Green’s function analysis method for each individual in the optimization tool. The optimization begins by accepting the desired steering angles and generating randomly initialized solutions for the bias voltages to form the first population. As the search evolves, the population includes fitter solutions that are transferred to the next generation through the tournament selection procedure along with the crossover or mutation operators. In the crossover, offspring populations are generated by combining two solutions with the best fitness values (parents) from the previous population space. However, the mutation introduces random changes into characteristics of the individuals. The offspring and parent population are then combined to form an extended population. This process is repeated iteratively until the results are converged to the global Pareto front solutions. The inverse design algorithm aims to minimize the deviations of the phase gradient across the perimeter-controlled array from the desired phase profile of the individually biased metasurface. In the mean time, the directivity, which is the measure of directional capabilities of metasurface and depends on both phase gradient and amplitude variations, is maximized. Therefore, non-intuitive distributions for the amplitude and phase profiles of the perimeter-controlled reflectarray are obtained that are simultaneously taken into account in calculating the optimal solution space. To accommodate for the large dimension of the input parameter space, the population size is adjusted to 200. The other options such as the creation, crossover, and elite functions are set to the defaults in the MATLAB R2019a optimization toolbox for the multi-objective GA solver. The optimization is terminated when the user-defined stop criteria is satisfied and the Pareto optimal front population is extracted, whose fraction is specified to 0.35 of the population size.

**Figure 4:**
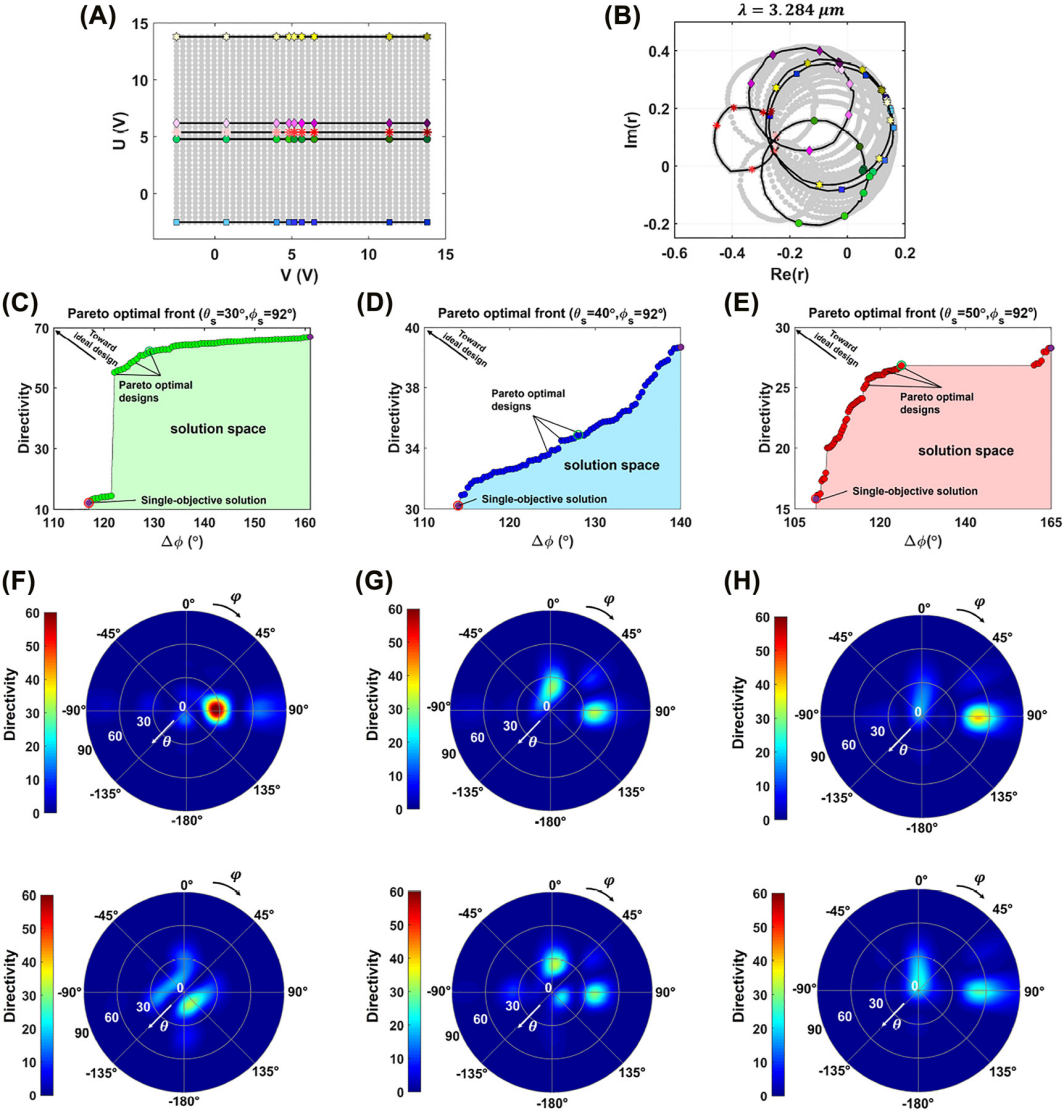
Inverse design of the perimeter-controlled beam steering metasurface. (A) The DC voltage space consisting of all possible combinations of the bias voltages *V* and *U* that are independently applied to the top and bottom electrodes. (B) The distribution of the reflection coefficient at the complex *r*-plane at the operating wavelength of *λ* = 3.284 μm that are resulted by application of the bias voltage combinations in (A) to the metasurface. The highlighted trajectories in (B) correspond to the color-coded lines at the bias voltage space in (A). Depiction of the solution space generated by multi-objective optimization when the steering angle of *φ*_*s*_ is adjusted to 92° and *θ*_s_ is set to the values (C) 30°, (D) 40°, and (E) 50°. The horizontal and vertical axes denote the deviation of the desired spatial phase from the obtained one and the directivity, respectively. The corresponding directivity patterns of the perimeter-controlled metasurface that are calculated for the steering angles (F) *θ*_s_ = 30°, *φ*_s_ = 92°, (G) *θ*_s_ = 40°, *φ*_s_ = 92°, and (H) *θ*_s_ = 50°, *φ*_s_ = 92°. In (F)–(H), the figures at the top and bottom rows respectively correspond to the multi-objective optimizer solutions that are identified by green circles and single objective optimizer solutions depicted by red circles in the Pareto optimal fronts.

Figure 4(A) and (B) demonstrate the voltage space consisting of all possible combinations of *V* and *U* that are applied to the top and bottom gates of the metasurface and the resultant reflection phasors in the complex *r*-plane at the operating wavelength of *λ* = 3.284 μm. There is one-to-one mapping between the data in the reflection plane and any bias voltage combinations of (*V*, *U*) applied to the metasurface. To show this correspondence, we have identified five linear trajectories in the voltage space and their respective reflection phasors by the color-coded markers in Figure 4(A) and (B). The highlighted trajectories are for the cases when *U* is adjusted to −2.5 V (square markers), 4.8 V (circle markers), 5.4 V (asterisk markers), 6.2 V (diamond markers), and 13.8 V (hexagonal markers), while *V* is continuously changing from −2.5 to 13.8 V. The corresponding trajectories in the complex *r* − plane indicate an almost circular path, whose locations are shifting between the over- and under-coupled resonant regimes upon changing the bias voltage *U*. For the voltages up to *U* = 4.8, the circular path covers almost all four quadrants of the complex *r* − plane that provides full 2*π* phase span by variation of the *V* due to the spectral overlap between the two resonant modes supported by the metasurface unit cell. By altering *U* between 4.8 V to ≈6.2 V, the operating regime enters to the under-coupled state, in which the obtained phase span is substantially reduced. Upon further increment of *U* beyond 6.2 V, the resonant regime shifts back toward the over-coupled mode covering a two-dimensional region that provides all the achievable reflection coefficients shown by the grey dots in Figure 4(B). As the bias voltage increases form *V* = −2.5 V up to *V* = 13.8 V, the color of the markers on the circular trajectories in the phasor diagram turns from light to dark tones.

Once the data for the reflection coefficient of the tunable metasurface for all combinations of the bias voltages *V* and *U* is generated, we use it to calculate the optimal amplitude and phase of the individual elements in the perimeter-controlled array. As indicated earlier, the scattering response of every individual element in the perimeter-controlled reflectarray can be independently controlled by assigning two sets of bias voltages into its respective column and row. The multi-objective optimizer selects the input data, being the DC bias voltages of the columns and rows, from the 2 × 9 matrix, whose entries are continuously varying from −2.5 to 13.8 V. As such, all the possible solutions for the complex reflectivity of the tunable metasurface should be generated in advance in order to be used in optimization problem to evaluate the objective functions that the optimizer seeks to minimize. The input matrix dimensions are determined by the column and row counts of the array.

To evaluate the objective functions in Eq. (6), many instances of parallel multi-objective GA coupled with Green’s function analysis technique are used to generate a dataset of optimized active perimeter-controlled reflectarrays with optimal tradeoffs between the spatial phase distributions across the metasurface and its directivity, when the operating wavelength is adjusted to *λ* = 3.284 μm. Demonstrated in Figure 4(C)–(E) are the Pareto optimal fronts obtained by multi-objective GA when *φ*_s_ = 92° and *θ*_s_ accepts three different values of 30°, 40°, and 50°, respectively. It is observed that due to the tradeoff between the competing objectives, the minimal deviations of the spatial phase distribution from the desired one is obtained at the cost of reduced directivity. The plots show that directivity of the metasurface evolves over multiple iterations and the optimization is terminated when further improvement in the results is not achievable and the Pareto optimal fronts are converged. The solution space for any combinations of the input bias voltages lies below the Pareto optimal front for all steering angles, implying that the tradeoff between their phase distribution and directivity is suboptimal. The Pareto optimal solutions that enable the best tradeoffs between the objectives and have the minimal deviation from the ideal design (left top corner of the solution space) are identified by arrows in Figure 4(C)–(E). In addition, in order to provide a comparison against the multi-objective optimization, we have highlighted the single-objective solutions by the purple circle markers in Figure 4(C)–(E). It can be seen that the single objective optimizer cannot offer optimal design that simultaneously maximizes the directivity and minimizes spatial phase deviations. Indeed, it only concentrates on providing the optimal response for one of the objectives and as a result eliminates the solution space into two outputs corresponding to maximal *D*_0_ or minimal Δ*ϕ*. The multi-objective inverse design problems come with enhanced computation cost in comparison to the single-objective optimization due to the consideration of at least two simultaneous antenna-specific functional responses. For the problems analyzed in this work with the array count of 9 × 9, the computation time for each iteration of the multi-objective solver is approximately 60 min, while it is reduced to approximately 30 s for the single-objective GA. The optimal results are generally obtained within 40–50 and 400–600 generations of the multi-objective and single-objective optimizers, respectively. The optimization problems are analyzed on the Desktop machine with the processor specifications of Intel Xeon W-2225 CPU @ 4.10 GHz, 4 cores, and 8 logic processors.

As a representative case study, we have plotted the radiation pattern of the optimal perimeter-controlled quasi-static reflectarray for three points that are specified by green circles as Pareto optimal solutions in Figure 4(C)–(E) at the operating wavelength of *λ* = 3.284 μm and the results are illustrated in Figure 4(F)–(H) top row. Two-dimensional beam steering toward the angles (*θ*_s_, *φ*_s_) = (30°, 92°), (40°, 92°), and (50°, 92°) is enabled and the maximum directivities are calculated as 60.56, 36.83, and 33.85, respectively. As the steering angle of *θ*_s_ grows steeper, the directivity of the perimeter-controlled reflectarray is reduced, which is attributed to the increased impedance mismatch between the incident and reflected waves [66]. In addition, the power coupling into the undesired sidelobes is increased at the broadside direction for the case (*θ*_s_, *φ*_s_) = (40°, 92°) that is due to the large amplitude variations of the constituent elements. We remark that the directivity of the reflectarray can be further increased by integrating more elements and increasing the effective size of the aperture. The effective aperture size in this work is considered rather small around (≈3λ) in order to expedite the computational analysis.

To provide a comparative overview, the results of the beam steering perimeter-controlled reflectarray designed by the single-objective optimizer (specified by red circles in Figure 4(C)–(E)) are also calculated for the same steering angles and indicated in Figure 4(F)–(H), the bottom row. It can be clearly observed that the single-objective optimizer cannot succeed in generating beam steering results with the main beam pointing toward the intended steering angles due to the deviations of the spatial distributions of the phase across the metasurface from the ideal case (Figure 4(F) bottom row). Additionally, since the optimizer only takes the effect of spatial distribution into account, the directivity levels are substantially low compared to the multi-objective optimization that are calculated as 8.087, 30.15, and 26.19 at the steering angles (*θ*_s_, *φ*_s_) = (30°, 92°), (*θ*_s_, *φ*_s_) = (40°, 92°), and (*θ*_s_, *φ*_s_) = (50°, 92°), respectively. Furthermore, the radiation patterns are dominated by the power residing at the undesired directions leading to increased sidelobe levels (Figure 4(G), (H) bottom row). The diffraction efficiencies of the metasurface designs obtained by the multi-objective and single-objective optimizers are obtained and compared in Section S4 of the Supplementary Material.

**Figure 5:**
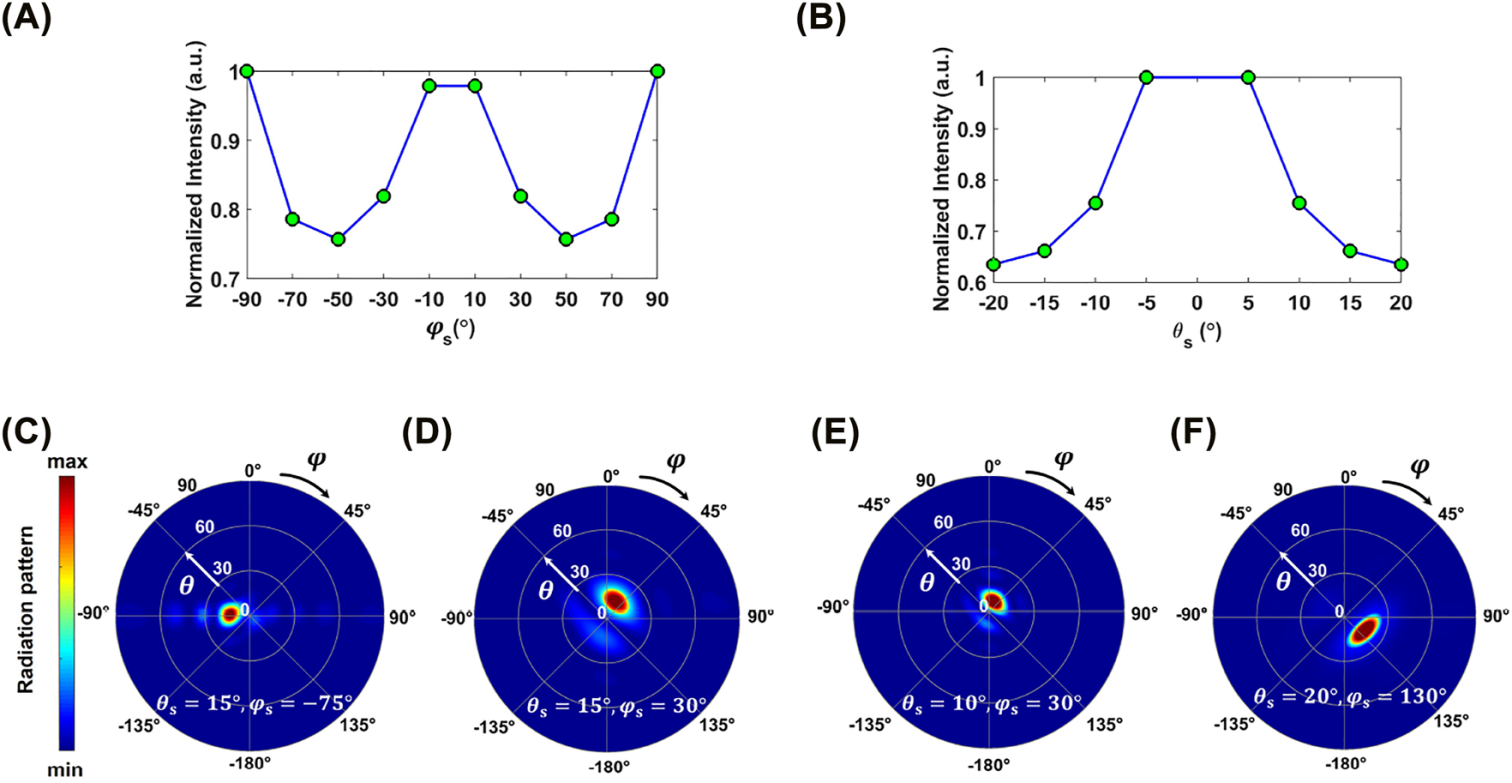
Perimeter-controlled beam steering with large FOV. The normalized intensity as a function of (A) *φ*_s_, when *θ*_s_ = 15° and (B) *θ*_s_, when *φ*_s_ = 30°. The normalized radiation patterns when the steering angles are (C) (*θ*_s_ = 15°, *φ*_s_ = -75°), (D) (*θ*_s_ = 15°, *φ*_s_ = 30°), (E) (*θ*_s_ = 10°, *φ*_s_ = 30°), and (F) (*θ*_s_ = 20°, *φ*_s_ = 130°).

To show the capability of our proposed perimeter-controlled approach for continuous beam steering with wide FOV, we further generalized it by calculating the radiation performance for larger data points being the steering angles along the azimuth and elevation directions. For this purpose, we have considered the same array configuration and studied two cases. First, *θ*_s_ is adjusted to 15° and *φ*_s_ is varying from −90° to +90° with the angular steps of 20°. Second, *φ*_s_ is selected as 30° and *θ*_*s*_ takes the values in the range of −20° to 20° with the angular increments of 5°. The far-zone radiation patterns are calculated and the normalized intensities at the desired steering angles are depicted in Figure 5(A) and (B). From the results one can see that the maximum intensities are obtained around the broadside direction and by moving away toward the azimuth angles of *φ*_s_ = 45°, the intensity reaches to its smallest level. This is attributed to the distribution of the phase across the perimeter-controlled array that is different from the ideal case of the individually-biased metasurfaces. However, by further increment of the *φ*_s_ toward the endfire direction, the field intensity is increased again. This is achieved thanks to the perimeter-controlled architecture of the bias voltages that are assigned to the columns and rows. To be specific, for 0° < *φ*_*s*_ < 45°, the row voltages play an essential role in defining the radiation pattern and adjusting the steering angles, while this is switched to the column voltages for 45° < *φ*_*s*_ < 90°. Therefore, the low performance is observed around 45°. Due to the symmetry, a similar plot as in Figure 5(A) can be attained for *φ*_*s*_ < −90° and *φ*_*s*_ > +90° covering the entire azimuth direction. It should be pointed out that although the optimization algorithm has significantly compromised the non-idealities of the perimeter-controlled array, the obtained elevation angles stray away from the target angles at the steeper directions. This can be confirmed from the two typical cases of the radiation patterns that are demonstrated in Figure 5(C) and (D) for *φ*_*s*_ = −75° and *φ*_s_ = +30°, respectively, when *θ*_s_ = 15°. In Figure 5(C), the target and obtained steering angles along both elevation and azimuth angles are identical; this is while in Figure 5(D) the obtained *θ*_s_ is 13° that deviates from the desired one for approximately 2°. This is the underlying reason for lower field intensity at *φ*_s_ = 30°–70° in Figure 5(A), as the intensity is calculated exactly at *θ*_s_ = 15°, while the obtained angle is about 2° off from the desired angle. In a similar fashion, for the second case, where the intensity plot as a function of *θ*_s_ is calculated, one can observe that beam steering with high efficiency is achieved up to the angle *θ*_s_ = 20° (Figure 5(B)). However, the intensity is reduced by moving away from the broadside direction. For steering angles beyond *θ*_s_ > 20°, the incident power is mainly coupled to the unwanted sidelobes leading into a reduction in the efficiency of the metasurface. The far-zone radiation pattern is plotted for two typical steering angles of (*θ*_s_, *φ*_s_) = (10°, 30°) as well as (*θ*_s_, *φ*_s_) = (20°, 130°) and the results are demonstrated in Figure 5(E) and (F). These findings highlight the applicability of the perimeter-controlled array in continuous beam steering from −20° to +20° and over the entire 360° along the elevation and azimuth angles, respectively. It should be mentioned that the steeper steering angles along the elevation direction (|*θ*_s_| > 20°) with high diffraction efficiency is obtained when the azimuth angle is set to *φ*_s_ = 0°, *φ*_s_ = 90°, *φ*_s_ = 180°, and *φ*_s_ = 270°. We have provided further information around the comparative study on the performance of individually-biased and perimeter-controlled arrays and the phase and amplitude distribution across the perimeter-controlled metasurface aperture at different steering angles in Sections S5 and S6 of the Supplementary Material.

From the results presented in Figures 4 and 5 that are obtained by inverse design algorithm, it can be concluded that by changing the steering angle of *θ*_s_ within the range −20° to +20°, two-dimensional continuous beam steering with full FOV along the azimuth angle can be dynamically acquired. Beam steering toward the angles |*θ*_s_| > 20° can be maintained by fixing the azimuth angle to 0°, 90°, 180°, or 270°. As such, the performance of the perimeter-controlled reflectarray for two-dimensional steering is reduced down to continuous beam steering along broadside and endfire directions for |*θ*_s_|> 20°. We should note that despite the advantages offered by perimeter-controlled biasing architecture, the limitations such as decreased directivity, deviations from the desired steering angles, and selective FOV along the elevation angle are imparted to its performance compared to the individually-biased metasurface. We have minimized the effect of such inherent drawbacks by employing the inverse design method.

## 4 Conclusions

In this article, we proposed a metasurface paradigm to realize a perimeter-controlled active reflectarray for dynamic two-dimensional beam steering at mid-infrared frequency regime. For this purpose, we integrated two active layers of InAs into the plasmonic patch nanoantennas in MIM configuration, and applied two sets of independent DC bias voltages to the gate electrodes for dynamically tailoring their refractive indices. Due to the low effective electron mass of the InAs layers, a wide phase span of around 355° is acquired by spatial modulation of the charge carriers within the active layers. The configuration of our proposed reflectarray allows for perimeter-control biasing architecture, in which the amplitude and phase of the individual elements can be modulated through bias voltage application to their respective columns and rows. The column-row addressing of the metasurface has facilitated the phase tuning in the individual element level that is maintained thanks to the independent control over two sets of biasing voltages in the dual-gated biasing network. Although our proposed perimeter-control biasing configuration surmounts the inherent complexity of the individually biased arrays composed of large number of densely-spaced unit cells, it suffers from reduced directivity, limited FOV along the elevation angle, and deviations of the desired steering angle from the realized one. Such limitations are partially overcome by using multi-objective evolutionary optimization algorithm for the inverse design of perimeter-controlled beam steering reflectarray and identifying the optimal tradeoffs between the spatial phase distribution across the entire array and its directivity. Despite the single-objective optimization algorithm, the multi-objective optimization yielded a subset of solutions with the best tradeoff between competing objectives, which provided the designer with an intuition into the ultimate boundary of the solution space. Our simulation results revealed that upon controlling the elements with the bias voltages assigned to corresponding columns and rows, two-dimensional beam steering with full (0° − 360°) and wide 40° FOV can be attained along the azimuth and elevation directions. Our proposed perimeter-controlled reflectarray platform enabled two-dimensional point scanning for any arbitrary combinations of the (*θ*_s_, *φ*_s_) with −20° ≤ *θ*_s_ ≤ 20°. The beam steering toward steeper angles of |*θ*_s_| > 20° is obtained by adjusting *φ*_s_ to 0°, 90°, 180°, and 270°. The simplified perimeter-control biasing architecture paves the way toward designing large-aperture metasurface composed of millions of elements in the array that are essential for the long-range operation.

## Supplementary Material

Supplementary Material Details
