# Supplementary material for: Inverse design of perimeter-controlled InAs-assisted metasurface for two-dimensional dynamic beam steering
Source: Nanophotonics. 2022 Sep 2;11(20):4515–30. doi: 10.1515/nanoph-2022-0376 (PMC9507428; doi:10.1515/nanoph-2022-0376)
Supplement: Supplementary file 1 — Supplementary Material Details [file j_nanoph-2022-0376_suppl.pdf]

Supplementary Material for "Inverse design of perimeter-controlled InAs-assisted metasurface for two-dimensional dynamic beam steering"

Raana Sabri and Hossein Mosallaei[1, a)]

Department of Electrical and Computer Engineering, Northeastern University, Boston, MA, 02115, USA

(Dated: 14 August 2022)

The Supplementary Material is organized in six sections:

S1. Device simulations and carrier dynamics

S2. Origin of the resonant mode at longer wavelength

S3. The cross coupling effects of the metasurface adjacent unit cells

S4. Diffraction efficiency calculation

S5. Spatial phase distribution comparison: individually biased v.s. perimeter-controlled reflectarrays

S6. The amplitude and phase distribution across the perimeter-controlled metasurface aperture for typical values of steering angles

a)Electronic mail: hosseinm@ece.neu.edu

S1. DEVICE SIMULATIONS AND CARRIER DYNAMICS

The carrier dynamic simulation of our proposed InAs-integrated plasmonic metasurface is carried out by using Lumerical device solver. In the device simulations, the spatial distributions of the charge carriers are modeled under the application of the bias voltages through the Poisson and drift-diffusion equations. The carriers (electrons and holes) move under the influence of drift due to the applied bias voltages and random thermal diffusion due to the gradient in the density. In the main manuscript, we remarked that the upper boundaries of the bias voltages are limited by the breakdown field strength of the alumina gate-dielectric that is reported as 7.4 MV/cm[1]. On the other hand, the lower threshold of the bias voltages is chosen in order to avoid the accumulation of the holes at the InAs/gate-dielectric interfaces. Figure S1(A)-(B) demonstrate the spatial distributions of the electrons and holes within the InAs active layer as functions of the top bias voltage of V, which is changing from -13.8 to 13.8 V. The bottom bias voltage U is adjusted to 0 V. From the distributions of the electrons one can see that by increasing the bias voltage from 0 V up to 13.8 V, the concentration of electrons are enhanced form $\approx 1 \times 10^{19}$ up to $\approx 13.8 \times 10^{19}$ at the InAs/alumina interface. The accumulated carrier densities exhibit an exponentially decaying profile by moving away from the InAs/alumina interface and the effective thickness of the accumulation layer becomes < 1 nm. Upon reducing the bias voltage toward negative values, the electron carriers are depleted from the interface. At the threshold voltage of $V_{\mathrm{T}} = -2.5$ V, the thickness of the depletion layer reaches to the maximum and beyond that the hole carriers are generated. The accumulation of the holes can be observed at the InAs/alumina interface for the bias voltages < -2.5 V in Fig. S1(B). The hole densities attenuate exponentially for larger distances from the interface. The accumulation of holes at InAs/alumina interface for the applied bias voltages $V < V_{\mathrm{T}}$ leads to a decrement in the real part of the permittivity of InAs, while giving rise to its imaginary part. As a result, the reflection amplitude and phase of the metasurface are reduced. To avoid the destructive impact of hole accumulation in the optical response of the metasurface, we have set the lower boundaries of the bias voltage into -2.5 V.

It should be noted that in order to generate the dataset demonstrated in Fig. 4(B) of the main manuscript, the charge carrier distributions for all possible combinations of the bias voltages V and U, that are continuously varying in the range of -2.5 V to $+13.8$ V, are maintained. However, for the sake of brevity, only the typical case in which U is adjusted to 0 V, is depicted. After generating the charge carrier distribution within the InAs active layer, its permittivity is calculated

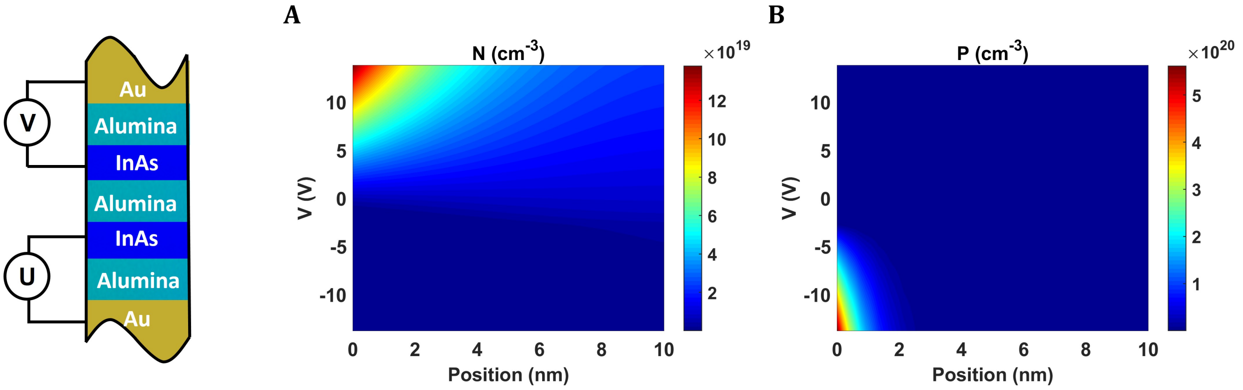

Fig. S1. Electrostatic representation of the InAs-integrated MIM configuration and dual-gated independent biasing mechanism that leads to charge accumulation/depletion at the interfaces. (A) Spatial distribution of the carrier density of the (A) electrons and (B) holes at the top InAs/alumina interface when V is changing from -13.8 to $+13.8$ V and U is adjusted to 0 V.

through the Drude dispersion model. Next, the electric permittivity is used to model the InAs in the RCWA simulations for generating the reflection coefficient dataset demonstrated in Fig. 4(B) of the main manuscript.

S2. ORIGIN OF THE RESONANT MODE AT LONGER WAVELENGTH

In this section, we further elaborate on the nature of the observed resonant mode at the longer wavelength in reflection profile of the InAs-assisted plasmonic metasurface. From Figs. 3(A)-(B) of the main manuscript, we note that the reflection spectrum is characterized by one reflection dip for the bias voltage of $V < 4$ V. As soon as the bias voltage is increased beyond 4 V, the permittivity of the InAs layer approaches to ≈ 0, and the second resonant mode at the longer wavelengths is excited. This resonant mode is associated to the epsilon-near-zero (ENZ) material and is referred to as the ENZ resonance. To investigate this behavior further, we performed FDTD simulations and calculated the metasurface response to the variations of the parameters namely bias voltage, collision frequency, thickness of the ENZ layer, and the width of patch nanoantenna and the results are summarized in Figs. S2(A)-(E). We note that when the electrode voltages of the unit cell are set to $(0, 0)$ V, there is only one resonance at the reflection spectrum that is attributed to the gap plasmon mode. By increasing the level of the bias voltage assigned to the top electrode into 4 V and 4.5 V, respectively, the permittivity of the accumulation layer within the top InAs

approaches to the ENZ regime ($-1 < \varepsilon_{\text{InAs}} < 1$), which results in the excitation of ENZ mode (Figs. S2(A)-(B)). This mode is heavily damped by the material loss. To verify, we calculated the optical response of the metasurface by increasing the collision frequency within the accumulation layer from 2×10^{13} to 1×10^{14}. While the gap plasmon resonance remains almost unchanged by injecting more dissipative loss into the ENZ thin film, the ENZ mode is substantially damped, as shown in Fig. S2(C). In addition, the ENZ wavelength is strongly dependent to thickness of the accumulation layer, while having a weak dependence on the antenna dimension. These are confirmed by varying the parameters h_{Acc} and W corresponding to the height of the ENZ layer and width of the patch antenna. As demonstrated in Fig. S2(D), by increasing the thickness of the ENZ layer from 1 nm to 5 nm, a large spectral shift of 165 nm is observed for the ENZ resonance, while the shift in the spectral position of the gap plasmon resonance is only 45 nm. Figure S2(E) shows the weak dependence of the ENZ mode into the nanoantenna width. Although the gap plasmon mode shifts to longer wavelengths by increasing the antenna width, the spectral position of the ENZ resonance remains unchanged.

To gain a further insight, the near-field distribution of the z component of the electric field is calculated at the resonant wavelengths 3.39 μm and 3.61 μm that correspond to the gap plasmon and ENZ modes, respectively. From the results demonstrated in Figs. S2(F)-(G) it is observed that at the shorter resonant wavelength, the light interacts with the patch antenna and is enhanced within all the ENZ layer, InAs layers and the gate-dielectrics. Since the ENZ wavelength is independent of the antenna dimension, the light interaction is weak with the patch antenna at the second resonance wavelength, while it is strongly enhanced within the top accumulation layer and the intensity of the electric field is at least three times larger than the gap plasmon mode, corresponding to an enhancement of approximately 50 (See Figs. S2(F)-(G)). Therefore, our results confirm that the second mode is associated to the ENZ resonance of the InAs accumulation layer. Our results regarding the nature of ENZ mode are consistent with the observations in Ref.[2], where the dependence of optical response of the dipole antenna on an ENZ substrate is illustrated.

S3. THE CROSS COUPLING EFFECTS OF THE ADJACENT UNIT CELLS

To study the mutual effects of the under-biased adjacent unit cells, we conducted FDTD simulations for a super cell that is composed of two InAs-assisted MIM unit cells, whose building block is demonstrated in Fig. 1(B) of the main manuscript. We have considered three cases

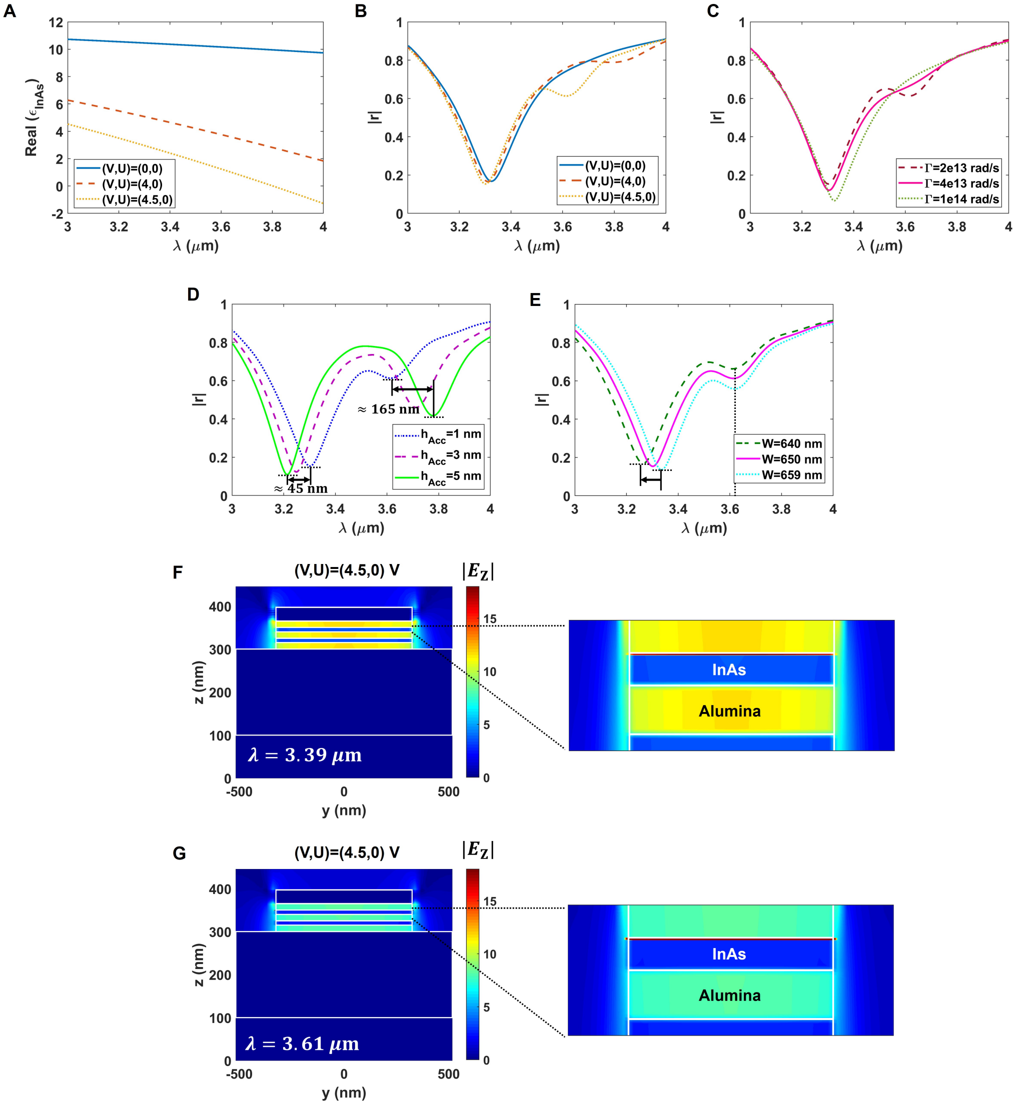

Fig. S2. (A) The real part of the permittivity of InAs as a function of wavelength for the bias voltage combinations of $(V,U) = (0,0)$ V, $(V,U) = (4,0)$ V, and $(V,U) = (4.5,0)$ V. The spectral response of the metasurface reflection coefficient by varying the parameters (B) bias voltages, (C) collision frequency, (D) thickness of the ENZ medium, and (E) width of the patch nanoantenna. The distributions of the z component of the electric field within the metasurface unit cell at the (F) gap plasmon and (G) ENZ wavelengths. The insets at the right sides of (F)-(G) demonstrate the field within InAs/alumina/InAs/alumina heterostructure.

for the configuration of the DC electrical biasing assigned to the constituent elements of the super cell and calculated the optical response. Cases I-III respectively correspond to the biasing voltages $[(V,U) = (0,0), (V,U) = (0,0)]$ V, $[(V,U) = (5,0), (V,U) = (5,0)]$ V, and $[(V,U) = (0,0), (V,U) = (5,0)]$ V, where the first and second entries of the brackets are applied to the left and right unit

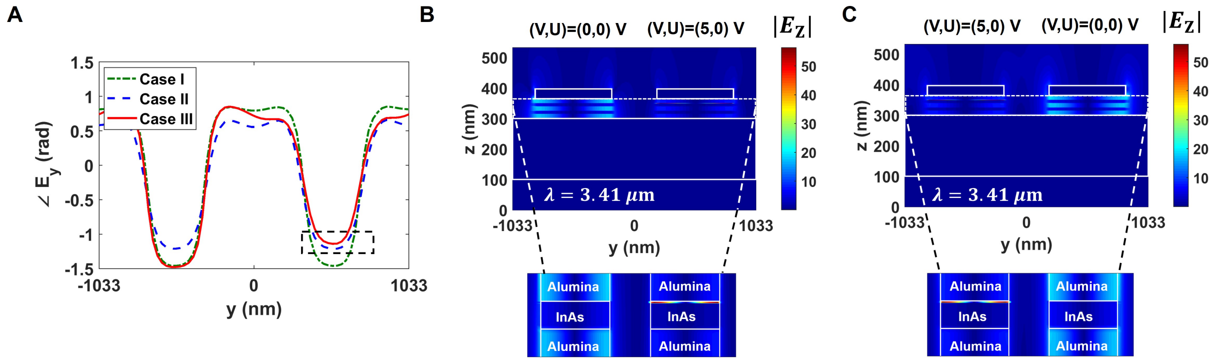

Fig. S3. (A) The phase profile of the reflected field immediately above the metasurface super cell, when the biasing configurations of the supercells are adjusted to $[(V,U)=(0,0),(V,U)=(0,0)]$ V, $[(V,U)=(5,0),(V,U)=(5,0)]$ V, and $[(V,U)=(0,0),(V,U)=(5,0)]$ V, corresponding to Cases I-III, respectively. The near-field distribution of the z component of the electric field within the metasurface super cell at the operating wavelength of $\lambda = 3.41$ μm when the biasing of the elements are adjusted to (B) $[(V,U)=(0,0),(V,U)=(5,0)]$ V and (C) $[(V,U)=(5,0),(V,U)=(0,0)]$ V. The insets of (B)-(C) show the zoomed-in view of electric field within the alumina/InAs/alumina spacer.

cells, respectively. Figure S3(A) demonstrates the phase of the electric field reflected from the metasurface super cell upon applying the bias voltage configurations in Cases I-III into the elements that is obtained immediately above the super cell. It is observed that the reflection phase over the left element in Case III overlaps with Case I, however there is a small deviation between the phase profiles of Case II and Case III over the right element that is denoted by the dashed black box in Fig. 3(A). This deviation which is smaller than 4° is attributed to the destructive effect of its adjacent unit cell. This implies that the crosstalk effects of the metasurface neighboring unit cells are negligible. It is worth mentioning that to further increase the device durability, the elements can be coated by a capping insulator layer that can further isolate the adjacent elements and minimize their side effects on each others performance as proposed in[3].

In addition, we have calculated the near-field distribution within the metasurface super cell to further investigate the influence of the electrical biasing upon one unit cell on the performance of its adjacent unit. We have studied Case III and its complementary scenario by switching the voltage configurations of the unit cells positioned at the left and right side of the super cell and the results are shown in Figs S3(B)-(C). It is observed that by applying the 5 V bias voltage into any of the unit cells, the accumulation layers are solely formed within the under biased unit

cell, without affecting the performance of the unbiased unit cell. By switching the bias voltage configuration to the Case demonstrated on top of Fig. S3(C), the accumulation layer is formed within the left unit cell while the element at its right side remains unaffected. The insets of Figs. S3(B)-(C) depict the close-up image of the distribution of the z component of the electric field within Alumina/InAs/Alumina spacer.

S4. DIFFRACTION EFFICIENCY CALCULATION

In Figs. 4(F)-(H) of the main manuscript, we have shown several examples of the beam steering device that is enabled by the inverse design of the perimeter-controlled reflectarray. It has been demonstrated that although the simplified biasing architecture by row-column addressing of the elements allows for beam deflection to the target angles, the incident power can be also coupled to the unwanted directions. The underlying reason can be related to the deviations of the perimeter-controlled array amplitude and phase from the desired array profiles. Nevertheless, from the results in the main manuscript (Figs. 4-5), it is clearly seen that the reflected beam can be steered over the range of $\pm 20^\circ$ along the elevation angle, while covering the entire 0° to 360° along the azimuth direction. The steering angles of $|\theta_s| > 20^\circ$ can be achieved when the azimuth angle is adjusted to $0^\circ, 90^\circ, 180^\circ$ and 270°. In order to quantify the performance of our proposed perimeter-controlled reflectarray, we have calculated the diffraction efficiency for the directivity radiation patterns demonstrated in Figs. 4(F)-(H) of the main manuscript and the results are depicted in Fig. S4 by the bar graphs. Here, the diffraction efficiency is defined by the ratio of the beam directivity in the intended region to the sum of the directivity at all bright spots in the plotted radiation patterns[4]. Figures S4(A)-(C) show the far-zone radiation patterns calculated by the multi-objective GA for the cases where the target steering angles are adjusted to $(\theta_s, \varphi_s) = (30^\circ, 92^\circ)$, $(\theta_s, \varphi_s) = (40^\circ, 92^\circ)$, and $(\theta_s, \varphi_s) = (50^\circ, 92^\circ)$, respectively. Demonstrated in Figs. S4(D)-(F) are the same cases optimized by the single-objective solver. The diffraction angles excited by all six designs are identified via the rectangles over the radiation patterns. The red and blue rectangles correspond to the desired and undesired diffraction angles at each case, respectively. The diffraction efficiencies for the designs performed by multi-objective GA are calculated as 72.91%, 30.15%, and 39.39% for the cases (A)-(C) at the intended regions, which are substantially larger compared to the efficiency at the undesired diffraction angles (shown at the bottom rows of the Figs. S4(A)-(C)). On the other hand, the diffraction efficiencies of the beam

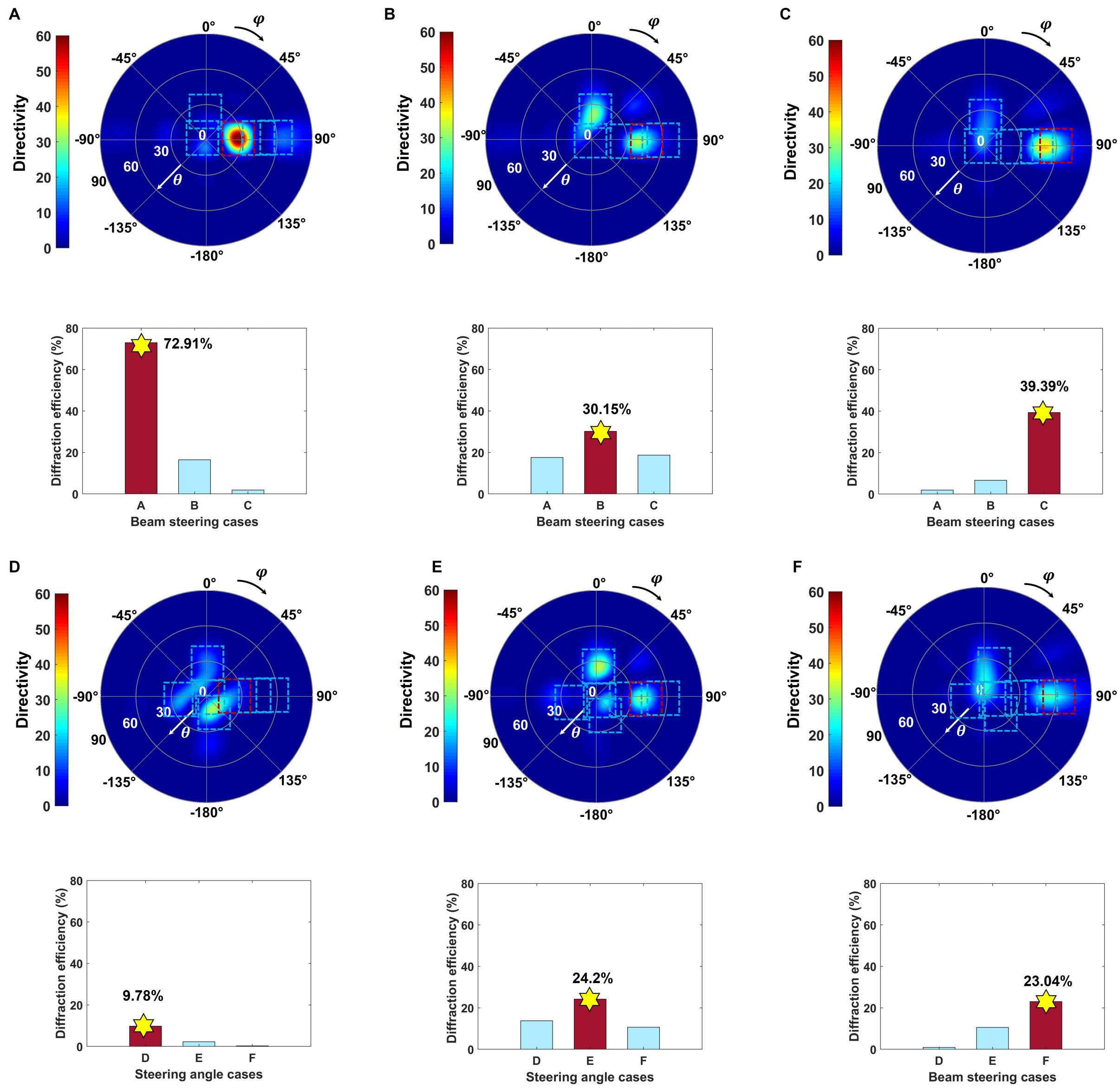

Fig. S4. Diffraction efficiency calculation. Far-field radiation patterns for the designs obtained by the multi-objective GA when the steering angles target to (A) $(\theta_s, \varphi_s) = (30°, 92°)$, (B) $(\theta_s, \varphi_s) = (40°, 92°)$, (C) $(\theta_s, \varphi_s) = (50°, 92°)$. The bottom rows of (A)-(C) correspond to the diffraction efficiency bar plots, respectively. The maximum diffraction efficiencies of 72.91%, 30.15%, 39.39% at the intended angles are achieved for the cases (A)-(C). (D)-(F) same as (A)-(C) for the designs obtained by single-objective optimizer. The maximum diffraction efficiencies in this case are calculated as 9.78%, 24.2%, 23.04% at the intended angles corresponding to (D)-(F). The red and blue rectangles in (A)-(F) top rows correspond to the intended and unintended steering angles, respectively. The diffraction efficiency at the desired regions are identified by the pentagonal markers in (A)-(F) bottom rows.

steering arrays designed by the single-objective GA are obtained as 9.78%, 24.2%, and 23.04% at the target angles of cases (D)-(F), respectively (illustrated at the bottom row of Figs. S4(D)-(F)). It is clear that the diffraction efficiencies of the metasurface designs obtained by the multi-objective GA are larger compared to the single-objective solver, verifying the capability of multi-objective optimizer in maximizing the directivity which is defined as the second objective. From the results in Figs. S4(D)-(F) (bottom row), one can see that although the single-objective solver leads to less efficient designs compared to its multi-objective counterpart, it still has a good performance in maximizing the diffraction efficiency at the target steering angles compared to the efficiencies of the undesired sidelobes.

S5. SPATIAL PHASE DISTRIBUTION COMPARISON: INDIVIDUALLY-BIASED V.S. PERIMETER-CONTROLLED REFLECTARRAYS

The implementation of two-dimensional beam steering metasurface requires simultaneous and independent control over its constituent unit cells. Such controlling technique can be obtained by either individual biasing of the elements or the perimeter-control architecture. For the long-range communication, the large-aperture arrays are required, where individual biasing of their constituent elements presents a significant challenge. The perimeter-controlled architecture simplifies the biasing mechanism of the large-aperture arrays by reducing the control signals to $2N$ through addressing the corresponding rows and columns. In this section, we have performed a comparative study on the spatial distribution of the amplitude and phase of the perimeter-controlled and individually-controlled reflectarrays and their resultant radiation patterns. For this purpose, we have employed multi-objective GA for generating the desired far-zone radiation patterns and the amplitude and phase profiles of the perimeter-controlled reflectarray, while the forward approach is utilized for designing the individually-controlled metasurface. The reflectarray is composed of an ensemble of 9×9 unit cells that are arranged along the x and y directions, where the spacing between the elements is adjusted to Λ. Figure S5 shows two examples of beam steering that are obtained by the two biasing configuration (individually- and perimeter-controlled architectures). For the forward design of the individually-controlled reflectarray, we have used the data that provides an almost 2π phase span with the constant reflection amplitude level of ≈ 0.15 for the metasurface unit cell. Such data for the reflection is achievable and can be confirmed from the reflectivity result at the complex r-plane demonstrated in Fig. 4(B) of the main manuscript. By

proper selection of the bias voltage combination applied to the dual-gated metasurface, a phasor diagram resembling a circle can be inscribed to the complex r-plane that can provide a constant amplitude of ≈ 0.15 due to the equal distance from the origin and complete 2π phase shift. In Figs. S5(A)-(D), a comparison between the array profiles of the perimeter-controlled and individually-biased metasurfaces is provided when the steering angles are adjusted to (θ_s, φ_s)=($35°, 0°$) and (θ_s, φ_s)=($16°, 84°$). For the first case, it can be seen that the spatial distributions of the phase for the perimeter-controlled (Fig. S5(A) middle row) and the individually-biased (Fig. S5(B) middle row) arrays are identical. As a result, the main beam generated by both reflectarrays are directed to the precisely same steering angles (Figs. S5(A)-(B) top rows). However, from the directivity radiation patterns (Fig. S5(A) top row) one can observe a power coupling to the specular reflection direction of the perimeter-controlled reflectarray. This is due to the non-uniform amplitude distribution across the metasurface along different columns. For the second case, by comparison of the results in Figs. S5(C)-(D) (middle and bottom rows) one can see that although the spatial phase and amplitude distributions of the perimeter-controlled reflectarray deviate from the desired case of the individually biased metasurface, the beam steering toward the target angles of (θ_s, φ_s)=($16°, 84°$) can be successfully obtained. This is achieved thanks to the multi-objective inverse design method that optimizes the array configuration toward attaining high spatial resolution of beam steering device and enhanced directivity by providing non-intuitive array and phase profiles of the perimeter-controlled reflectarray.

S6. THE AMPLITUDE AND PHASE DISTRIBUTION ACROSS THE PERIMETER-CONTROLLED METASURFACE APERTURE FOR TYPICAL VALUES OF STEERING ANGLES

In this section, we have shown beam steering into three typical cases in which the steering angles are adjusted to $(\theta_s, \varphi_s) = (8°, -68°)$, $(16°, 125°)$, and $(55°, 180°)$ and the results are shown in Figs. S6(A)-(C). The operating wavelength is set at $\lambda = 3.284$ μm and the reflectarray consists of 9×9 unit cells with the element spacing of 1033 nm. The spatial phase and amplitude distributions across the reflectarray are respectively depicted at the middle and right columns of Figs. S6(A)-(C). It can be seen that although the spatial distributions of the acquired phase across the perimeter-controlled reflectarray strays away from the desired patterns for the individually biased elements, the dynamic beam steering toward the desired directions with high diffraction efficiency

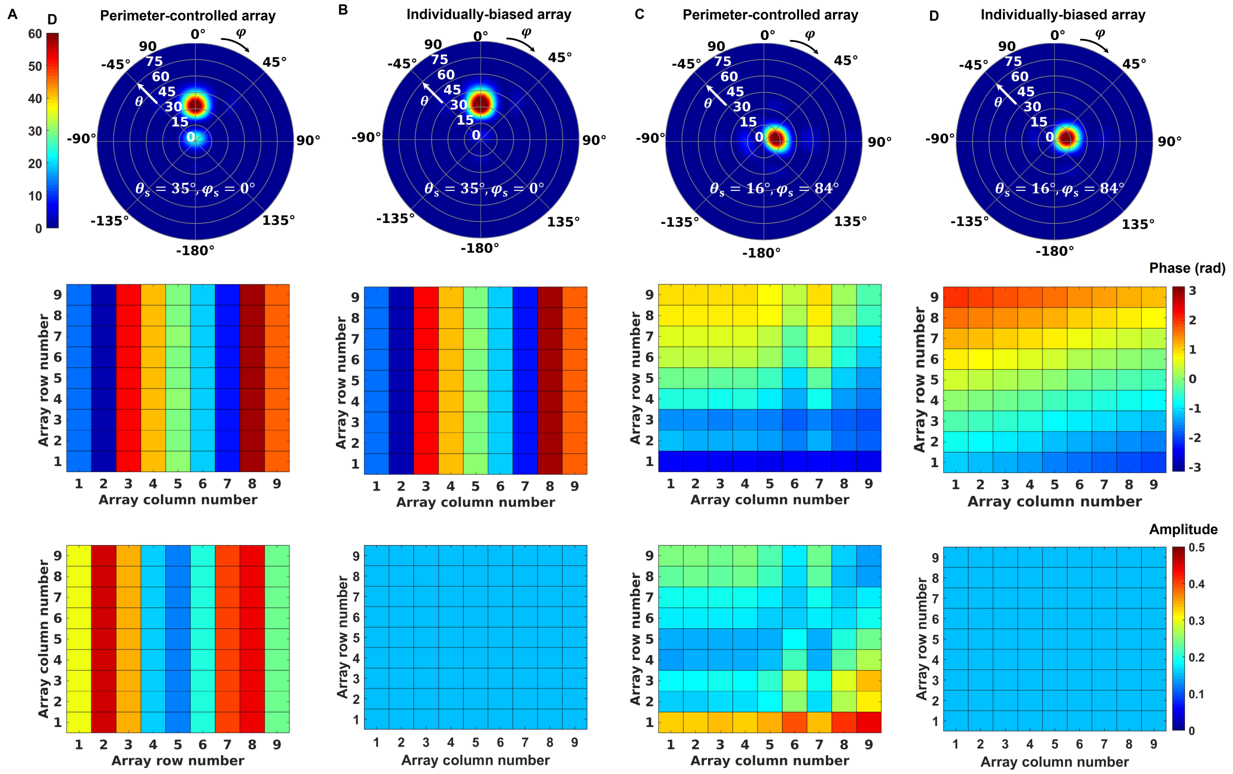

Fig. S5. The directivity radiation pattern comparison of the perimeter-controlled and individually-controlled reflectarrays and their corresponding phase and amplitude distributions. The steering angles are adjusted to (A)-(B) $(\theta_s, \varphi_s) = (35°, 0°)$ and (C)-(D) $(\theta_s, \varphi_s) = (16°, 84°)$.

can be still maintained. In addition, due to the covarying amplitude and phase response of unit cells, uniform amplitude for all the constituent elements of the reflectarray cannot be obtained. However, in our optimization problem, we optimize toward the maximum achievable directivity, therefore the algorithm aims to minimize the amplitude modulation over the entire array. This results in reduced sidelobe levels, as well. Moreover, the full FOV along elevation direction cannot be attained. The DC voltage profiles for addressing the columns and rows of the beam steering perimeter-controlled reflectarray are illustrated across their corresponding amplitude profiles, whose values are changing within the range -2.5 V to 13.8V. From Fig. S6 it can be concluded that the perimeter-controlled reflectarray allows for simplified biasing architecture and enables two dimensional dynamic beam steering with wide FOV along the azimuth and elevation angles, respectively. The inverse design technique based on multi-objective GA outputs nonintuitive amplitude and phase profiles for the perimeter-controlled array that succeeds in generating high resolution radiation patterns with enhanced directivity and reduced sidelobe levels.

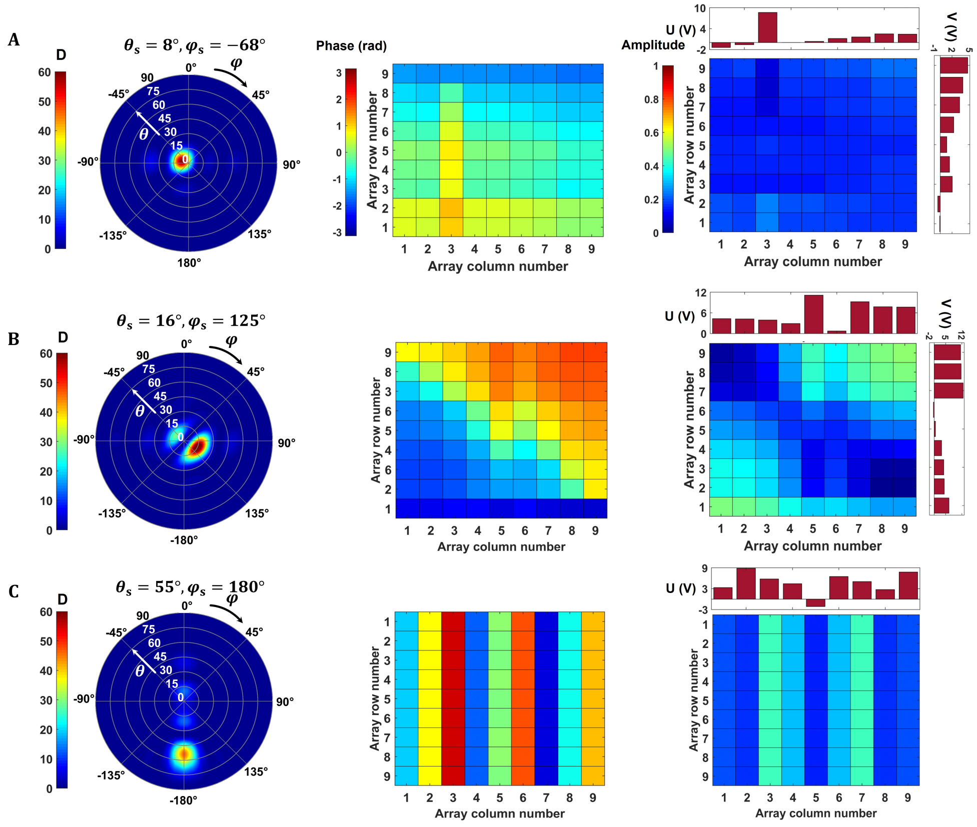

Fig. S6. The directivity radiation pattern comparison of the perimeter-controlled and individually-controlled reflectarrays and their corresponding phase and amplitude distributions. The steering angles are adjusted to (A)-(B) $(\theta_s, \varphi_s) = (35°, 0°)$ and (C)-(D) $(\theta_s, \varphi_s) = (16°, 84°)$.

REFERENCES

[1]G. Kafaie Shirmanesh, R. Sokhoyan, R. A. Pala, and H. A. Atwater, "Dual-gated active metasurface at 1550 nm with wide (> 300) phase tunability," Nano letters **18**, 2957–2963 (2018).

[2]S. A. Schulz, A. A. Tahir, M. Z. Alam, J. Upham, I. De Leon, and R. W. Boyd, "Optical response of dipole antennas on an epsilon-near-zero substrate," Physical Review A **93**, 063846 (2016).

[3]P. Thureja, G. K. Shirmanesh, K. T. Fountaine, R. Sokhoyan, M. Grajower, and H. A. Atwater, "Array-level inverse design of beam steering active metasurfaces," ACS nano **14**, 15042–15055 (2020).

[4]S. I. Kim, J. Park, B. G. Jeong, D. Lee, K.-Y. Yang, Y.-Y. Park, K. Ha, and H. Choo, "Two-dimensional beam steering with tunable metasurface in infrared regime," Nanophotonics **11**, 2719–2726 (2022).
